# The *IFITM5* mutation in osteogenesis imperfecta type V is associated with an ERK/SOX9-dependent osteoprogenitor differentiation defect

**DOI:** 10.1172/JCI170369

**Published:** 2024-06-17

**Authors:** Ronit Marom, I-Wen Song, Emily C. Busse, Megan E. Washington, Ava S. Berrier, Vittoria C. Rossi, Laura Ortinau, Youngjae Jeong, Ming-Ming Jiang, Brian C. Dawson, Mary Adeyeye, Carolina Leynes, Caressa D. Lietman, Bridget M. Stroup, Dominyka Batkovskyte, Mahim Jain, Yuqing Chen, Racel Cela, Alexis Castellon, Alyssa A. Tran, Isabel Lorenzo, D. Nicole Meyers, Shixia Huang, Alicia Turner, Vinitha Shenava, Maegen Wallace, Eric Orwoll, Dongsu Park, Catherine G. Ambrose, Sandesh C.S. Nagamani, Jason D. Heaney, Brendan H. Lee

**Affiliations:** 1 Department of Molecular and Human Genetics, Baylor College of Medicine, Houston, Texas, USA.; 2 Texas Children’s Hospital, Houston, Texas, USA.; 3 Medical Scientist Training Program, Baylor College of Medicine, Houston, Texas, USA.; 4 Medical Scientist Training Program, UT Health Houston MD Anderson Cancer Center, Houston, Texas, USA.; 5 Department of Orthopaedic Surgery, McGovern Medical School at UT Health, Houston, Texas, USA.; 6 Department of Molecular and Cellular Biology, and Huffington Department of Education, Innovation, and Technology, Advanced Technology Cores, and; 7 Department of Orthopedic Surgery, Baylor College of Medicine, Houston, Texas, USA.; 8 Orthopaedic Surgery, University of Nebraska Medical Center, Children’s Hospital and Medical Center, Omaha, Nebraska, USA.; 9 Department of Medicine, Bone and Mineral Unit, Oregon Health and Science University, Portland, Oregon, USA.

**Keywords:** Bone biology, Genetics, Bone development, Bone disease, Cartilage

## Abstract

Osteogenesis imperfecta (OI) type V is the second most common form of OI, distinguished by hyperplastic callus formation and calcification of the interosseous membranes, in addition to the bone fragility. It is caused by a recurrent, dominant pathogenic variant (c.-14C>T) in interferon-induced transmembrane protein 5 (*IFITM5*). Here, we generated a conditional *Rosa26-*knockin mouse model to study the mechanistic consequences of the recurrent mutation. Expression of the mutant *Ifitm5* in osteo-chondroprogenitor or chondrogenic cells resulted in low bone mass and growth retardation. Mutant limbs showed impaired endochondral ossification, cartilage overgrowth, and abnormal growth plate architecture. The cartilage phenotype correlates with the pathology reported in patients with OI type V. Surprisingly, expression of mutant *Ifitm5* in mature osteoblasts caused no obvious skeletal abnormalities. In contrast, earlier expression in osteo-chondroprogenitors was associated with an increase in the skeletal progenitor cell population within the periosteum. Lineage tracing showed that chondrogenic cells expressing the mutant *Ifitm5* had decreased differentiation into osteoblastic cells in diaphyseal bone. Moreover, mutant IFITM5 disrupted early skeletal homeostasis in part by activating ERK signaling and downstream SOX9 protein, and inhibition of these pathways partially rescued the phenotype in mutant animals. These data identify the contribution of a signaling defect altering osteo-chondroprogenitor differentiation as a driver in the pathogenesis of OI type V.

## Abstract 2

Disruptions to osteo-chondroprogenitor cell differentiation due to activated ERK/SOX9 signaling contributes to the abnormal skeletal development in osteogenesis imperfecta type V.

## Graphical abstract

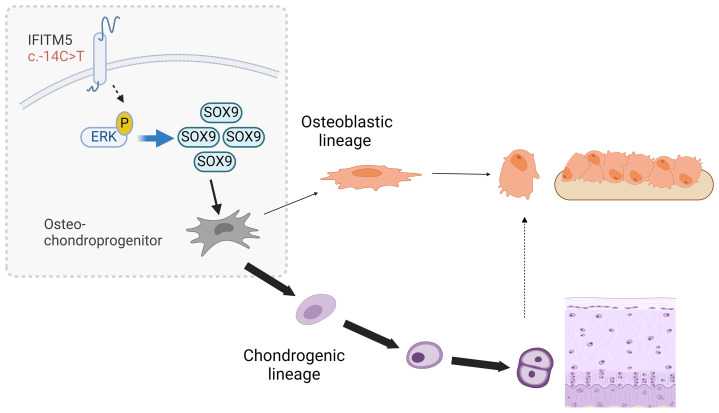

## Introduction

Osteogenesis imperfecta (OI) is an inherited multi-systemic disorder characterized by low bone mass, recurrent fractures, growth deficiency, and skeletal deformities (1–3). Extraskeletal manifestations may include muscle weakness, dentinogenesis imperfecta, hearing loss, and pulmonary disease (1–3). OI is genetically and phenotypically heterogenous. Most cases of OI (~85%) are caused by pathogenic variants in *COL1A1* or *COL1A2*, which encode the α 1 and α 2 chains of type I collagen, respectively, whereas the remainder are caused by pathogenic variants in genes involved in collagen processing or in the regulation of bone homeostasis (1, 3, 4). OI type V is the most common type of OI that is not caused by alterations in type I collagen and is estimated to account for up to 9%–10% of moderate-to-severe OI (5–7). Clinically, OI type V is characterized by the unique features of hyperplastic callus formation (which can occur with or without a fracture), and calcification of the interosseous membranes in addition to bone fragility (8). Notably, all individuals with OI type V have the same autosomal dominant pathogenic variant (c.-14C>T) in *IFITM5* (9–12). This variant resides in the 5′ untranslated region (5′-UTR) and introduces a new in-frame start codon, adding 5 amino acid residues at the N-terminus of the protein (13). *IFITM5* encodes interferon-induced transmembrane protein 5 (also known as BRIL), a member of the highly conserved IFITM (IFN-induced transmembrane) family of proteins that are implicated in the innate immune response (9–12, 14). Unlike other IFITM proteins that are expressed ubiquitously, *IFITM5* is primarily expressed in skeletal tissues, in osteoblasts (15, 16), chondrocytes (9, 15, 17), and progenitor niches like the groove of Ranvier (9). Studies suggest that it has a role in bone mineralization (15) but does not exhibit antiviral activity or other functions related to the immune system, as observed with other IFITM proteins (18).

The molecular consequences of the recurrent (c.-14C>T) OI type V pathogenic variant remain unknown. Expression of the mutant *Ifitm5* allele in transgenic mice overexpressing the mutant protein in bone, and in CRISPR/Cas9 knockin mouse models, both resulted in a severe OI phenotype with perinatal lethality (19–21). Importantly, overexpression of wild type *Ifitm5* and complete loss-of-function models showed no obvious phenotypes (16, 19). Thus, a neomorphic effect in bone has been suggested as the most likely mechanism. However, the cellular and signaling mechanisms by which the mutant IFITM5 drives the skeletal pathology are not understood. Here, we report a conditional *Rosa26* mutant *Ifitm5*-knockin mouse model that is viable and recapitulates the human phenotype, thus facilitating the understanding of the role of the mutant protein in specific stages of osteoblast and chondrocyte differentiation. Findings in this model, supported by studies in the previously reported transgenic *Ifitm5^c.-14C>T^* mice (19), as well as in zebrafish and in vitro cell culture models, point to downstream activation of extracellular signal–regulated kinase/mitogen-activated protein kinase (ERK/MAPK) signaling and increased SOX9 protein, leading to the abnormal osteo-chondroprogenitor differentiation in OI type V pathogenesis.

## Results

### Conditional expression of the mutant Ifitm5^c.-14C>T^ allele in limb mesenchyme (Rosa26^mIfitm5^ Prx1-Cre), but not in mature osteoblasts (Rosa26^mIfitm5^ OC-Cre), results in low bone mass.

**Figure 1:**
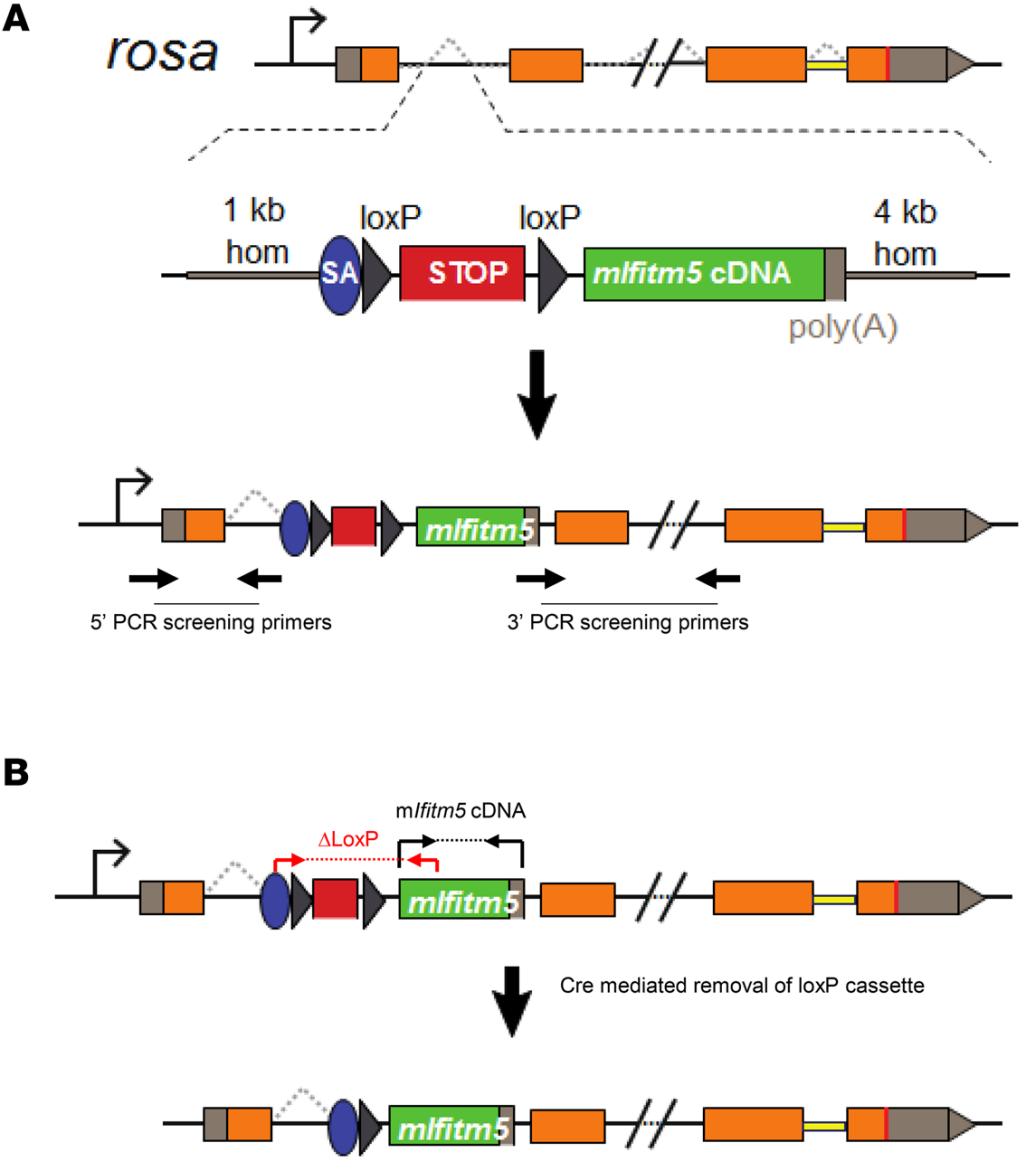
Conditional *Rosa26* knockin m*Ifitm5* mouse model. The mutant mouse *Ifitm5* cDNA was cloned into the *Rosa26*-DEST vector downstream of a loxP-polyA-loxP stop cassette. The schematic representation also indicates the approximate location of the PCR screening primers in ES cells (**A**) and genotyping primers in mice (**B**).

All mouse models generated to date for OI type V exhibit perinatal lethality. Therefore, we generated a conditional mouse expressing the disease-causing *IFITM5* mutation at specific stages of osteoblast and chondrocyte cell differentiation. We inserted the mutant *Ifitm5^c.-14C>T^* allele (m*Ifitm5*) into the *Rosa26* locus (22) via homologous recombination (Figure 1, A and B). To study whether the human OI type V phenotype is recapitulated in this model, we crossed mice that were heterozygous for the *Rosa26*-knockin m*Ifitm5* allele (*Rosa26*^mIfitm5/+^) with: (a) paired related homeobox 1 (Prx1-Cre) transgenic mice that expressed the Cre recombinase (Cre/–) in mesenchymal precursor cells of the osteo-chondroprogenitor lineage, and (b) osteocalcin-Cre (OC-Cre) transgenic mice that expressed the Cre-recombinase (Cre/–) in mature osteoblasts. Controls included littermates that expressed only the Cre/–, that carried only the *Rosa26*-knockin allele (*Rosa26*^mIfitm5/+^), or that carried neither allele (*Rosa26^+/+^*). The activation of *Cre* recombinase was validated by PCR using primers spanning the deleted *LoxP* cassette, and by real-time quantitative PCR (RT-qPCR) showing expression of the mutant *Ifitm5* mRNA in bone (Supplemental Figure 1; supplemental material available online with this article; https://doi.org/10.1172/JCI170369DS1).

**Figure 2:**
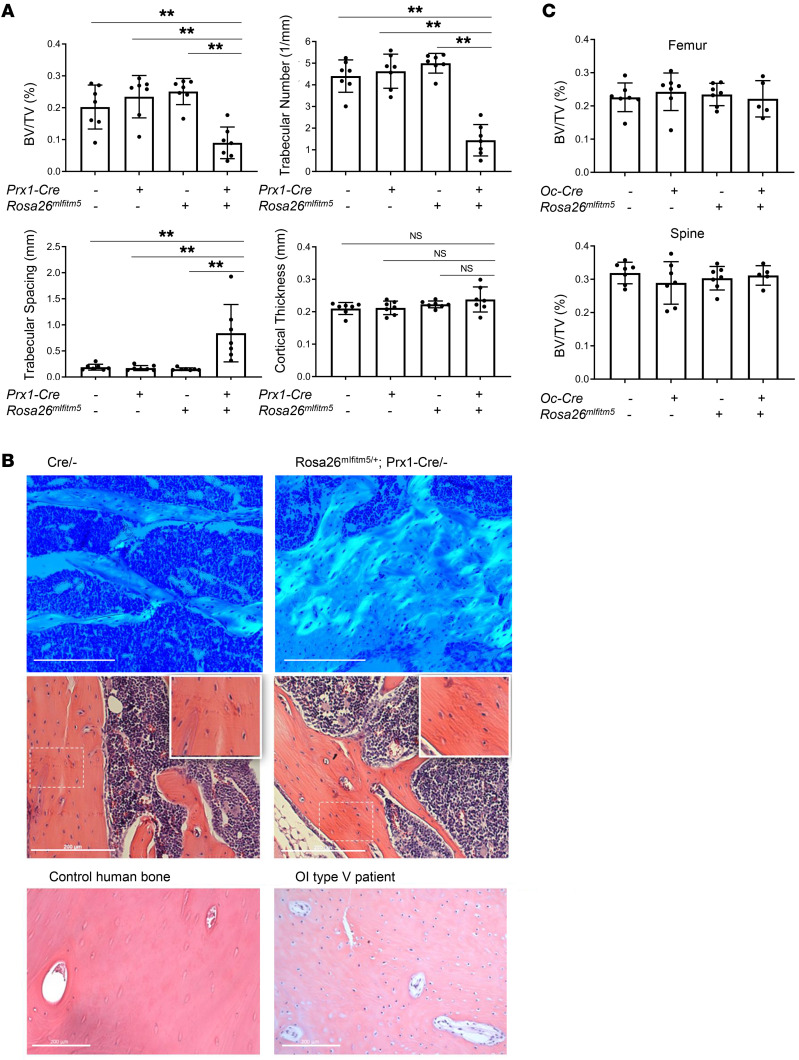
Conditional expression of the mutant *Ifitm5* allele results in low bone mass in *Rosa26^mIfitm5/+^* Prx1-Cre mice. (**A**) Micro CT analysis in femurs showed a significant decrease in bone architectural parameters in *Rosa26^mIfitm5/+^* Prx1-Cre/– mutant mice, including the BV/TV and trabecular number. Consistently, trabecular spacing was increased. Cortical thickness was not significantly altered. Analysis was performed in 2-month-old male mice (**P < 0.001, by 1-way ANOVA with Tukey’s post hoc tests; *n* = 7 per group; all comparisons were with the mutant group). (**B**) Histological sections show immature mesh-like bone matrix in *Rosa26^mIfitm5/+^* Prx1-Cre/– mice, as seen in individuals with OI type V. Images show toluidine blue and H&E staining of bone sections from *Rosa26^mIfitm5/+^* Prx1-Cre/– mice and littermate controls (upper and middle panels). Bottom panel shows a human OI type V bone section for comparison. Scale bars: 200 μm. Original magnification, ×20 (insets). See supplemental Figure 10 for staining control (secondary antibody). (**C**) Micro CT analysis of femur and spine BV/TV shows no significant difference in *Rosa26^mIfitm5/+^* Oc-Cre/– mice. Analysis was performed in 2-month-old male mice (1-way ANOVA with Tukey’s post hoc tests resulted in no significant differences; *n* = 5–7 per group; all comparisons were with the mutant group).

At age 2 months, trabecular bone volume to total volume (BV/TV), as measured by micro CT in distal femurs, was significantly reduced in the *Rosa26^mIfitm5^* Prx1-Cre mice in both males (Figure 2A) and females (Supplemental Figure 2). Consistent with this finding, the trabecular number was decreased and trabecular spacing was increased in *Rosa26^mIfitm5^* Prx1-Cre mice compared with littermate controls. Cortical thickness was not significantly affected, but histological sections showed immature bone, as seen in bone histology from a patient with OI type V (Figure 2B), and consistent with previous reports (8). Interestingly, expression of the mutant *Ifitm5* allele in mature osteoblasts in Rosa*26^mIfitm5^* OC-Cre mice did not have an obvious effect on bone mass in mutant mice (*Rosa26*^mIfitm5/+^ OC-Cre/–) compared with controls (Figure 2C and Supplemental Figure 3). We further studied the effect of mutant *Ifitm5* expression in committed, early-differentiating osteoblasts via the 2.3 kb rat Col1a1-Cre promoter (*Rosa26^mIfitm5^* Col1a1-Cre). In this model, the bone mass in mutant animals was not significantly different than in Cre controls (Supplemental Figure 4). The divergent observations in *Rosa26^mIfitm5^* OC-Cre and *Rosa26^mIfitm5^* Col1a1-Cre versus *Rosa26^mIfitm5^* Prx1-Cre mouse models represent the consequences of distinct temporo-spatial expression of the mutant protein and suggest an early developmental effect in osteo-chondroprogenitors, and that its expression in committed and mature osteoblasts is not a major contributor to the bone phenotype.

### Conditional expression of the mutant Ifitm5 allele results in abnormal cartilage development, leading to growth restriction and progressive joint deformity in Rosa26^mIfitm5^ Prx1-Cre mice.

**Figure 3:**
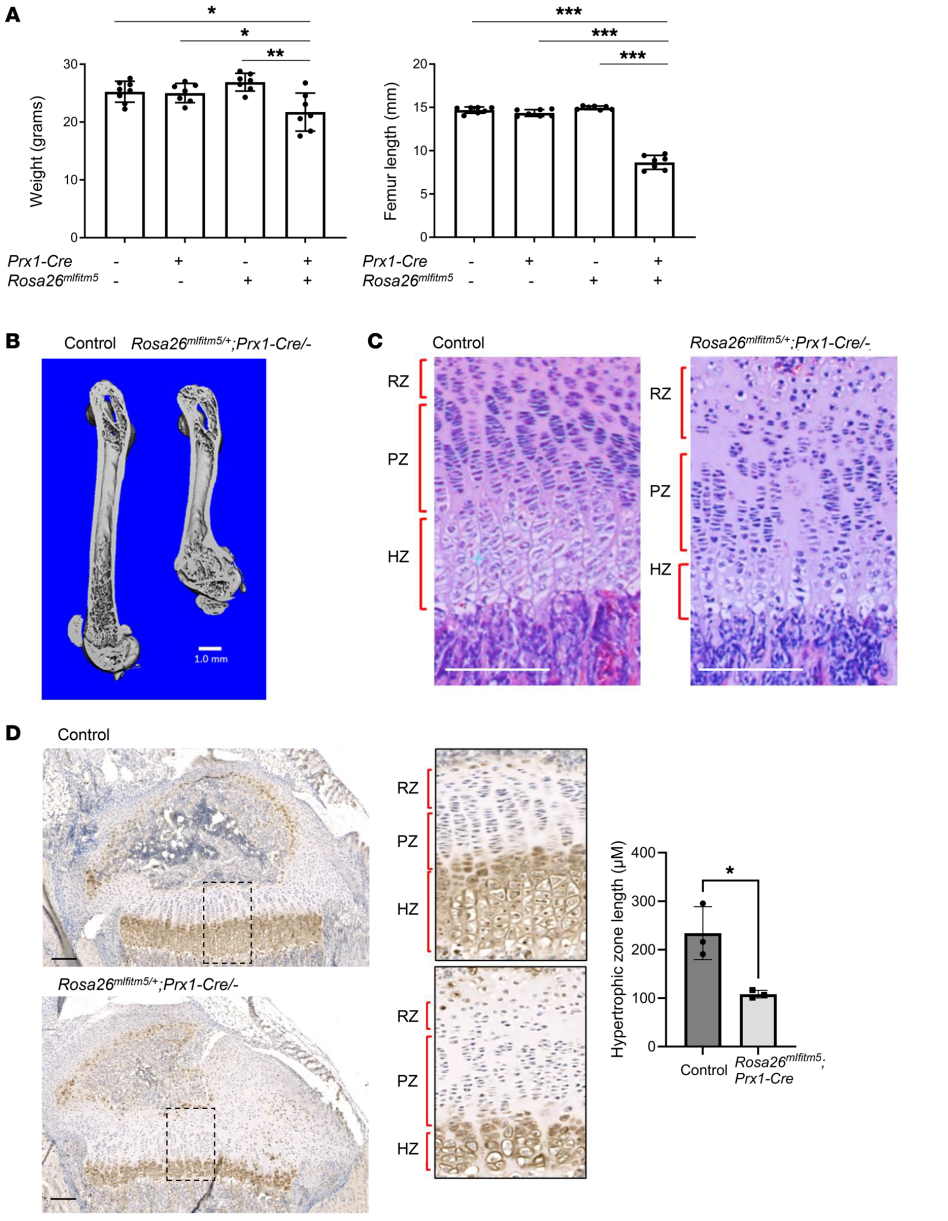
Conditional expression of the mutant *Ifitm5* allele results in growth restriction and abnormal growth plate architecture in *Rosa26^mIfitm5/+^* Prx1-Cre mice. (**A**) Body weight and femur length were reduced by 10%–15% and 40%–50%, respectively, in *Rosa26^mIfitm5/+^* Prx1-Cre/– mutant mice (a summary of measurements in 2-month-old male mice, 1-way ANOVA with Tukey’s post hoc tests; *n* = 7 per group, all were comparisons with mutant group. *P < 0.05, **P < 0.001, and ***P < 0.0001). (**B**) Representative micro CT image of a control (left) and mutant (right) mouse femur. (**C**) Representative H&E-stained histology images of the proximal tibia from control (left panel) and mutant (*Rosa26^mIfitm5/+^* Prx1-Cre/–, right panel) mice at 2 weeks of age. Images show that staggering of the proliferating chondrocyte was disrupted and the hypertrophic zone was shorter in the mutant mice. (**D**) IHC for type X collagen highlights the reduced hypertrophic zone (HZ) in mutant mice (*Rosa26^mIfitm5/+^* Prx1-Cre/–, bottom) at 2 weeks of age (right panel: insert magnified), and a summary of the hypertrophic zone length measurements (*P = 0.016, by 2-tailed Student’s *t* test; *n* = 3 per group). HZ, hypertrophic zone; PZ, proliferative zone; RZ, resting zone. Scale bars: 200 μm. Original magnification, ×20 (insets).

*Rosa26^mIfitm5^* Prx1-Cre–mutant mice showed reduced weight gain and significantly shortened femurs as compared with littermate controls (Figure 3, A and B, and Supplemental Figure 2). Histological sections of the tibial proximal growth plate at P14 showed disorganization of the columnar structure of the proliferative and hypertrophic zones in the mutant mice. Most notably, the hypertrophic zone was reduced, as demonstrated by IHC staining for type X collagen, a marker of hypertrophic chondrocytes (Figure 3, C and D).

**Figure 4:**
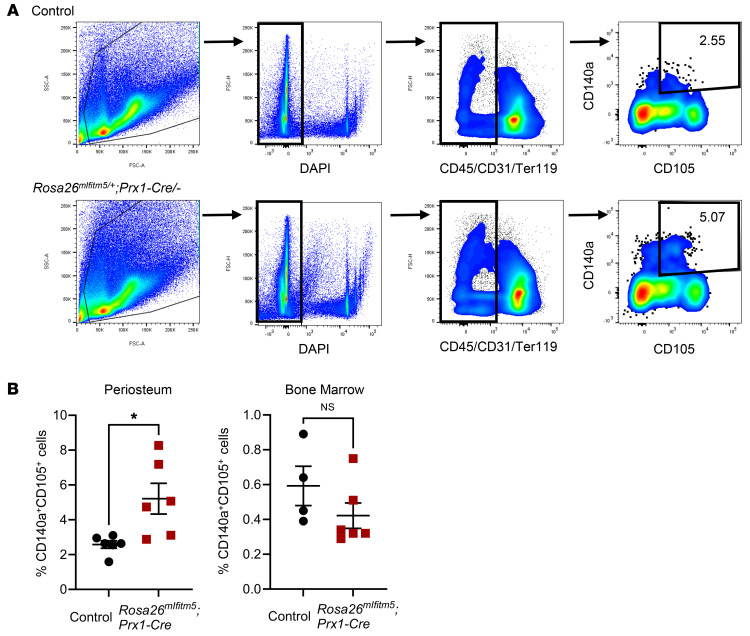
Conditional expression of the mutant *Ifitm5* allele results in increased periosteal skeletal progenitor populations in *Rosa26^mIfitm5/+^* Prx1-Cre mice. (**A**) Flow cytometric analysis of skeletal progenitor markers in cells isolated from the periosteum. (**B**) A significant increase in the CD105^+^CD140a^+^ cell population in *Rosa26^mIfitm5^* Prx1-Cre–mutant mice was detected in the periosteum but not in the bone marrow (*P = 0.015, by 2-tailed Student’s *t* test; *n* = 6 per group for periosteal cells; *n* = 4–6 per group for bone marrow cells).

Studies have shown that *Prx1-*expressing cells in the periosteum serve as a reservoir for osteo-chondroprogenitor cells during bone development and fracture healing (23, 24). Therefore, we next tested whether shortened femurs in the *Rosa26^mIfitm5^* Prx1-Cre–mutant mice were due to the defect of periosteal progenitor cell populations or to the defect of their differentiation. We performed flow cytometric analysis of periosteal skeletal progenitor cells by negative selection for hematopoietic (CD45), endothelial (CD31), and erythroid (Ter119) cell lineage markers and positive selection for CD105 and CD140a (25). Notably, this analysis demonstrated a significant increase in the percentage of the periosteal CD45^–^CD31^–^Ter119^–^CD105^+^CD140a^+^ cell population in *Rosa26^mIfitm5^* Prx1-Cre–mutant mice (5.07%) compared with littermate controls (2.55 %) (Figure 4, A and B), whereas the skeletal progenitor cell population in the bone marrow was not different (Figure 4B and Supplemental Figure 5). These data further support the effect of the OI type V mutation on skeletal progenitors as a potential driver of the phenotype.

**Figure 5:**
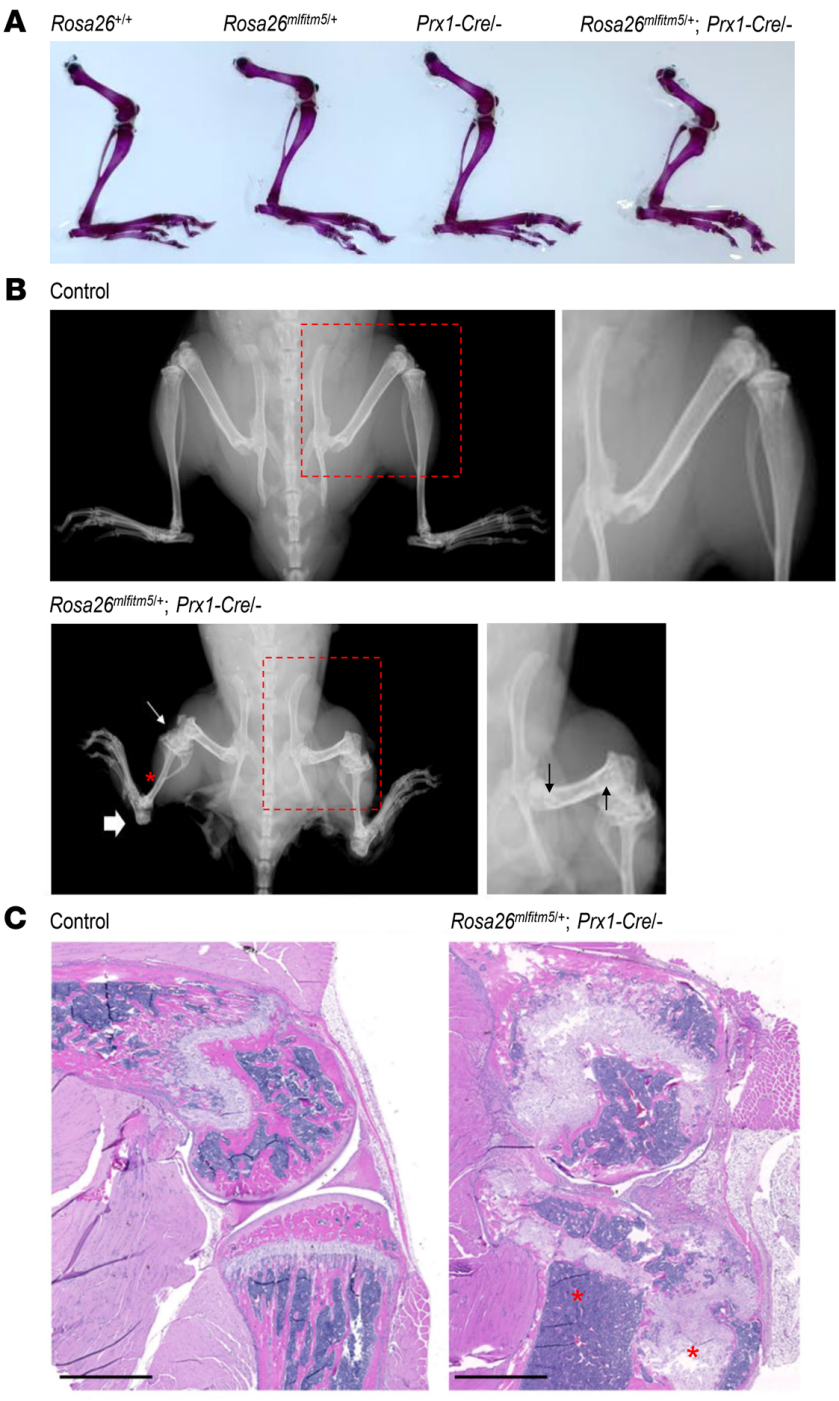
Conditional expression of the mutant *Ifitm5* allele in *Rosa26^mIfitm5/+^* Prx1-Cre mice leads to progressive cartilage overgrowth and abnormal development of the ossification centers. (**A**) Skeletal preparations of 3-week-old *Rosa26^mIfitm5/+^* Prx1-Cre/– mutant and littermate controls showing skeletal deformities including shortening and bowing of the femur and tibia, and partially mineralized cartilage overgrowth at the knee and ankle. (**B**) Skeletal radiographs at age 2 months demonstrating short and gracile long bones (red asterisk) with ossification around the knee and ankle (thin and thick white arrows, respectively). Magnified inserts on the right demonstrate sclerotic lines at the femur metaphysis (black arrows). (**C**) Representative H&E-stained histology images of the proximal tibia of control (left panel) and mutant (*Rosa26^mIfitm5/+^* Prx1-Cre/–, right panel) mice at 2 months of age, showing cartilage overgrowth and disruption of the endochondral ossification (red asterisks). Scale bars: 2 mm.

Skeletal preparations at P21 showed an irregular structure of the proximal long bones, with cartilaginous material accumulating in periarticular tissues (Figure 5A and Supplemental Figure 6). At age 2 months, skeletal radiographs revealed partial joint ossification, leading to joint deformities and contractures at the knees and ankles (Figure 5B). Histological analysis revealed progression of the abnormal growth plate ultrastructure, with cartilage overgrowth and disruption of endochondral ossification at the proximal tibial metaphysis (Figure 5C). In contrast, we observed that growth was not significantly altered in the *Rosa26*^mIfitm5/+^ OC-Cre/– or in *Rosa26^mIfitm5/+^* Col1a1-Cre/– mice that express the mutant allele in more mature osteoblasts (Supplemental Figures 3 and 4).

### Increased ERK signaling contributes to an abnormal skeletal phenotype in OI type V models.

**Figure 6:**
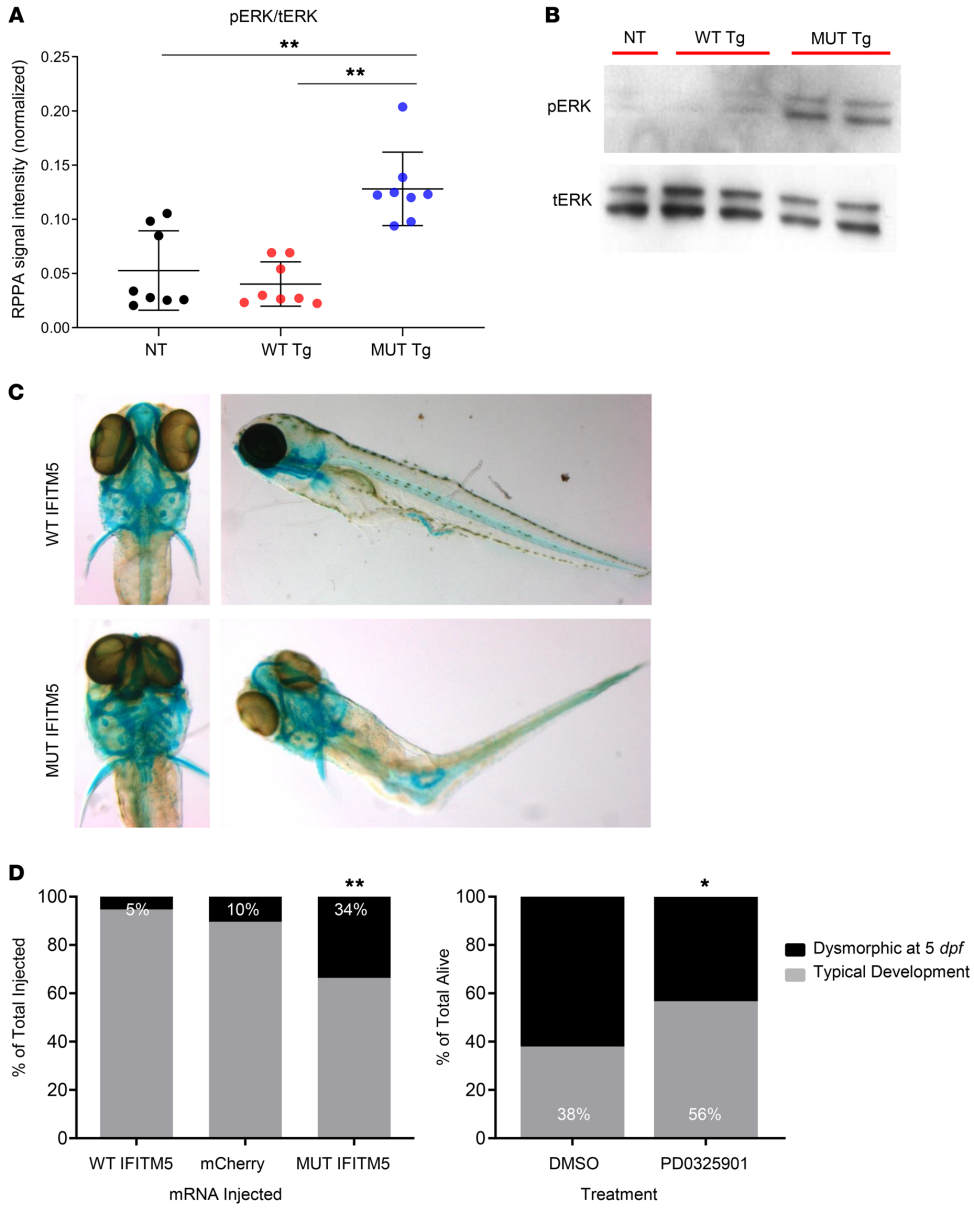
Activation of ERK signaling contributes to the abnormal skeletal development in OI type V. (**A**) RPPA results from the transgenic *Ifitm5^c.-14C > T^* mouse model. The ratio of pERK to total ERK (tERK) RPPA signal intensity was increased in calvaria protein extract from mice overexpressing mutant *Ifitm5* (MUT Tg), as compared with mice overexpressing wild type *Ifitm5* (WT Tg), and nontransgenic (NT) littermates (*n* = 8 per group, 1-way ANOVA with Tukey’s post hoc tests, all comparisons were with the MUT Tg group, **P < 0.001). (**B**) Western blot of representative RPPA samples for pERK and tERK in the transgenic *Ifitm5^c.-14C > T^* mouse model. (**C**) Representative image showing abnormal morphology (abnormal craniofacial development and tail kinking) of embryos injected with mutant *IFITM5* mRNA (MUT IFITM5, lower panel) at 5 dpf, compared with normal development of control zebrafish larvae (WT IFITM5, upper panel). Surviving larvae were fixed at 5 dpf and stained with Alcian blue. (**D**) Left panel: The proportion of IFITM5 mutant zebrafish that demonstrated dysmorphology (34%) was significantly greater compared with wild type and mCherry controls (5%–10%) (**P < 0.001, by χ^2^ test, *n* = 113–116 per group). Right panel: Treatment with the ERK inhibitor PD325901 (0.2 μM) partially rescued the abnormal development in IFITM5 mutant zebrafish. The proportion of IFITM5 mutant zebrafish with typical development increased by 18% at 5 dpf (*P < 0.05, by χ^2^ test, *n* = 97 in treatment group and *n* = 87 in the vehicle-treated group).

To study the molecular consequences of the IFITM5 mutation in bone, we performed proteomics analysis in calvaria of our previously reported transgenic mouse model that constitutionally overexpressed the mutant *Ifitm5* from the *Col1a1* 2.3 kb promoter (19). Reverse-phase protein array (RPPA) analysis detected elevated levels of phosphorylated ERK1/2 (pERK1/2) (Figure 6, A and B). This correlated with IHC staining for pERK in *Rosa*26^mIfitm5^ Prx1-Cre mice, which showed abundant positive staining in chondrocytes adjacent to the ossification centers at the proximal tibia (Supplemental Figure 7). To understand the functional consequences of increased ERK activation, we next generated an in vitro cell culture model of OI type V that stably overexpressed the mutant *Ifitm5* in MC3T3 cells under doxycycline induction. Consistent with the observations in OI type V mouse models, expression of mutant IFITM5 in MC3T3 cells was associated with increased ERK phosphorylation and delayed mineralization in vitro (Supplemental Figure 7). The delayed mineralization observed in this cell model was consistent with the effect on cultured calvarial osteoblasts in the previously published OI type V mouse models (19, 20). Importantly, treatment with the ERK inhibitor U0126 restored mineralization in MC3T3 *mIfitm5* mutant cells in a dose-dependent manner (Supplemental Figure 7).

To test the relationship between ERK activation and abnormal cartilage formation in OI type V, we generated a zebrafish (*Danio rerio*) model of OI type V and performed in vivo rescue experiments. Mutant *IFITM5* mRNA was injected into zebrafish embryos at the 1-cell stage (30 minutes to 1 hour post fertilization [hpf]). Subsequently, 34% of the embryos at 5 days post fertilization (dpf) showed abnormal morphology (abnormal craniofacial cartilage development and curved/kinked tail) compared with only 5%–10% in the control group zebrafish that were injected with either wild type *IFITM5* or *mCherry* mRNA (Figure 6, C and D). Treatment with the pharmacologic MEK/ERK inhibitor PD0325901 significantly increased by 18% the proportion of IFITM5 mutant zebrafish that survived with typical development at 5 dpf (Figure 6D).

### Altered spatiotemporal expression of SOX9 impairs chondrogenic maturation and disrupts endochondral bone formation in OI type V models.

**Figure 7:**
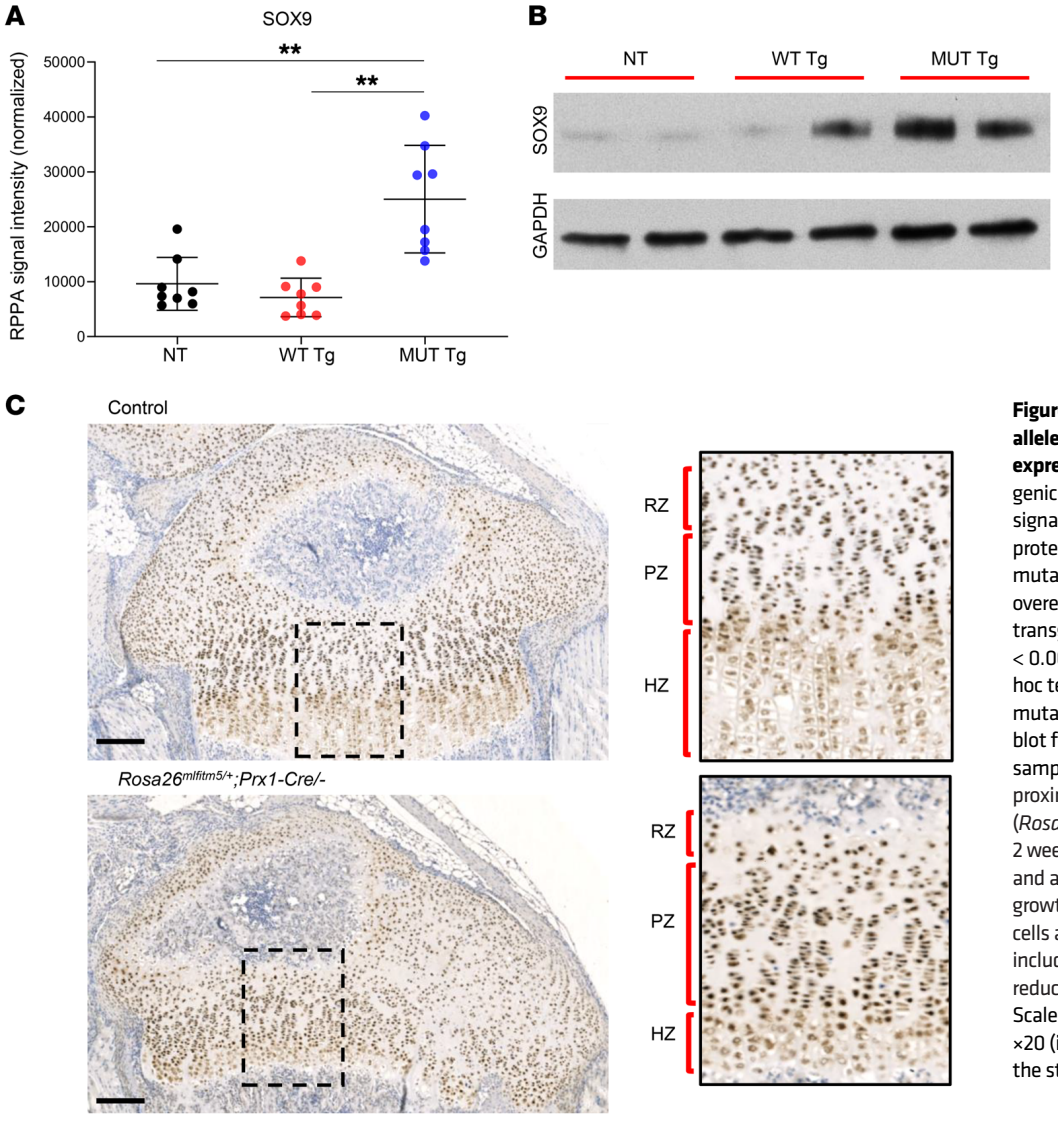
Expression of the mutant *Ifitm5* allele leads to altered spatiotemporal expression of SOX9. (**A**) RPPA in the transgenic *Ifitm5^c.-14C > T^* mouse model. SOX9 RPPA signal intensity was increased in calvaria protein extract from mice overexpressing mutant *Ifitm5*, as compared with mice overexpressing wild type *Ifitm5*, and nontransgenic littermates (*n* = 8 per group, **P < 0.001, by 1-way ANOVA with Tukey’s post hoc tests, all comparisons were with the mutant *Ifitm5* [MUT Tg] group). (**B**) Western blot for SOX9 in representative RPPA samples. (**C**) Representative IHC images of proximal tibia of control (top) and mutant (*Rosa26^mIfitm5/+^* Prx1-Cre/–, bottom) mice at 2 weeks of age, showing increased intensity and altered distribution of SOX9^+^ cells in the growth plate. In the mutant mice, SOX9^+^ cells are seen throughout the growth plate including the hypertrophic zone (which is reduced compared with littermate controls). Scale bar: 200 μm. Original magnification, ×20 (insets). See supplemental Figure 10 for the staining control (secondary antibody).

The RPPA analysis also detected increased levels of SOX9, a master regulator of cartilage development, in protein extracted from the mutant transgenic mice calvaria (Figure 7, A and B). Therefore, we studied the expression of SOX9 in conditional *Rosa26^mIfitm5^* Prx1-Cre–mutant mice that showed disruption of the growth plate structure and cartilage overgrowth (Figures 3 and 5). IHC for SOX9 in proximal tibial sections at P14 identified increased staining intensity and altered spatial distribution of SOX9^+^ cells in the growth plate, with persistence of SOX9 staining in hypertrophic chondrocytes (Figure 7C). Notably, Western blot analysis of ATDC5 chondrogenic cells demonstrated that wild type IFITM5 was coinduced with SOX9 expression during chondrogenic differentiation (Supplemental Figure 8).

**Figure 8:**
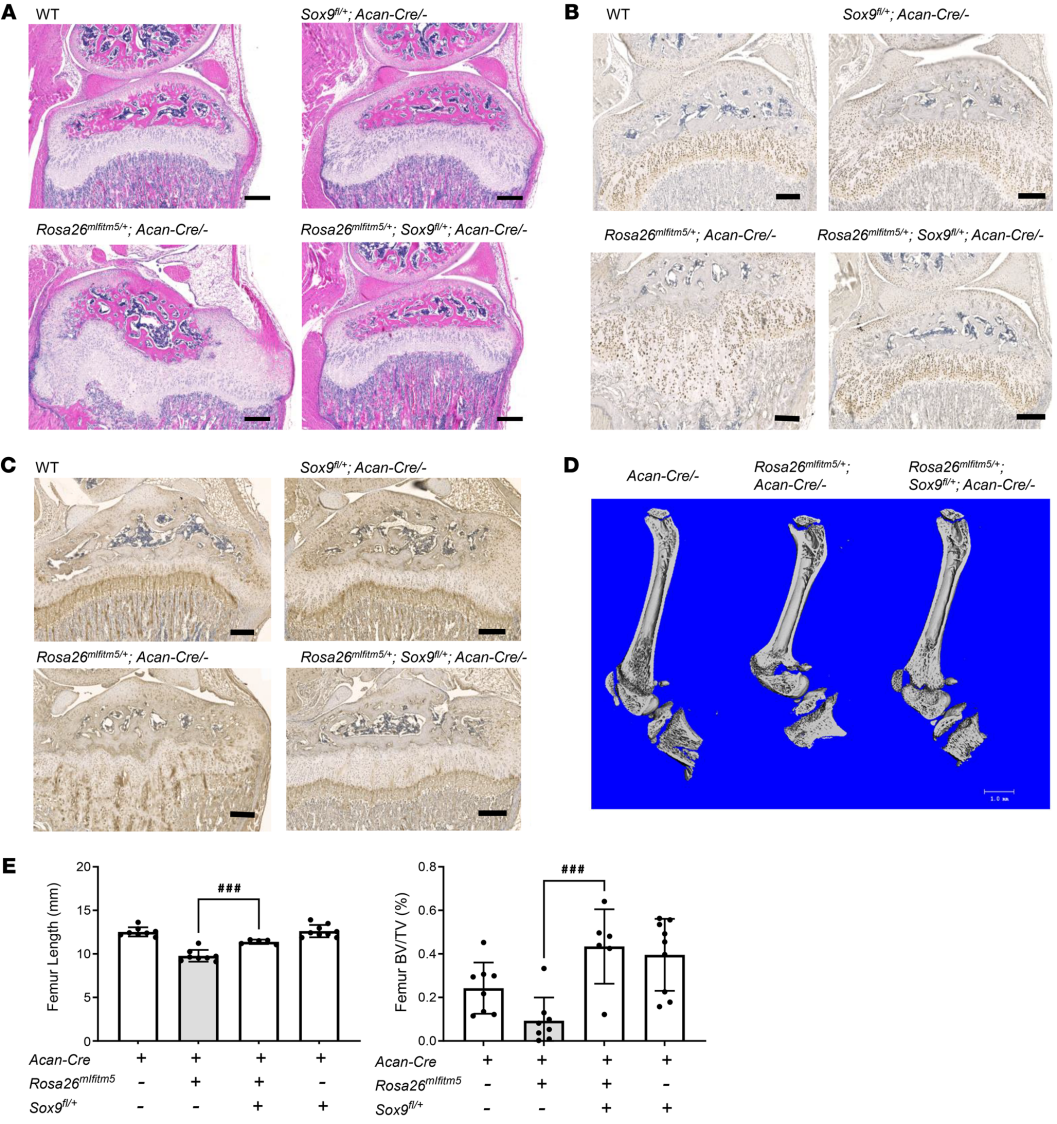
Abnormal skeletal development in *Rosa*26*^mIfitm5^* *Acan-Cre ERT2* mice is partially rescued by *Sox9* deletion. (**A**–**C**) Histology sections of *Rosa26*^mIfitm5/+^ *Acan-Cre* ERT2 mice, showing disrupted growth plate architecture caused by postnatal activation of mutant *Ifitm5* in chondrocytes (left panel, bottom), while *Sox9* deletion (right panel, bottom) rescued the growth plate structure. H&E staining (**A**) and IHC analysis for SOX9 (**B**) and type X collagen (**C**). Scale bars: 200 μm. (**D**) Images show growth delay in *Rosa26*^mIfitm5/+^ *Acan-Cre* ERT2–mutant mice (middle) compared with wild type (left) and partial rescue by *Sox9* deletion (right). (**E**) Micro CT analysis of femur length (left) and BV/TV (right) in *Rosa26*^mIfitm5/+^ *Acan-Cre* ERT2 mice, showing low bone mass in mutant mice and partial rescue by *Sox9* deletion (^###^*P* = 0.0005, by 1-way ANOVA, *n* = 6–8 per group). For all experiments shown, *Cre* recombinase was activated by i.p. tamoxifen injections (10 mg/kg/dose) at P10–P15, and samples were collected from mice at 5 weeks of age.

Prx1-Cre transgenic mice express the *Cre* recombinase in mesenchymal cells that differentiate into osteogenic and chondrogenic lineages. As mature osteoblastic lineage expression of mutant IFITM5 did not lead to abnormal skeletal phenotype, we explored whether cartilage-specific expression was sufficient to cause the skeletal phenotype. Thus, we studied the effect of postnatal activation of the mutant *Ifitm5* allele in chondrocytes via inducible *Rosa*26*^mIfitm5^* *Acan-Cre ERT2* mice. Similar to the findings in the Prx1-Cre mouse model, expression of mutant *Ifitm5* in chondrocytes under the aggrecan promoter at P10 disrupted the growth plate architecture, leading to disorganized columnar structure and a reduced hypertrophic zone, as shown by H&E staining and SOX9 IHC (Figure 8, A and B). Immunohistochemistry staining showed altered spatiotemporal expression of SOX9 and type X collagen in the mutant samples (Figure 8, B and C). Not surprisingly, *Rosa*26*^mIfitm5^* *Acan-Cre ERT2–*mutant mice exhibited a milder, but significant, growth defect and low bone mass, phenocopying the *Rosa26^mIfitm5^* Prx1-Cre mice (Figure 8, D and E). Moreover, biomechanical testing via 3-point bending showed reduced ductility and increased bone stiffness in *Rosa*26*^mIfitm5^* *Acan-Cre ERT2–*mutant mice (Supplemental Figure 9). To evaluate whether increased SOX9 is a driver of the skeletal phenotype, we intercrossed *Rosa*26*^mIfitm5^* *Acan-Cre ERT2* mice with *Sox9^fl^* mice. Postnatal genetic deletion of *Sox9* in cells expressing the mutant IFITM5 substantially improved the growth plate architecture, as shown by H&E staining and IHC for SOX9 and type X collagen (Figure 8, A–C). Furthermore, micro CT analysis demonstrated partial rescue of the growth restriction and low bone mass phenotype in *Rosa*26*^mIfitm5^* *Sox9^fl/+^* *Acan-Cre ERT2* mice (Figure 8E).

### OI type V is an osteo-chondrodysplasia.

**Figure 9:**
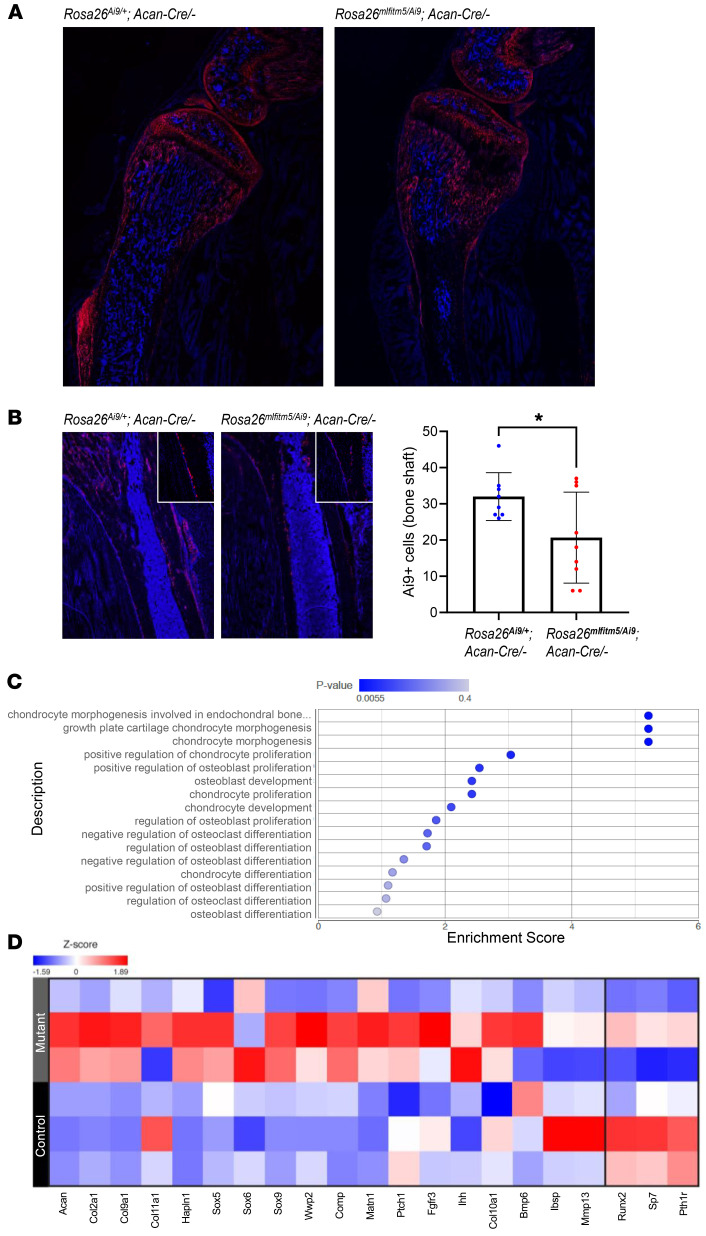
Expression of the mutant *Ifitm5* allele leads to altered skeletal stem cell differentiation and enhanced chondrogenesis. (**A**–**B**) Lineage tracing of chondrocytes coexpressing mutant *Ifitm5* and Ai9 reporter in *Rosa*26*^mIfitm5/Ai9^* *Acan-Cre ERT2* mice. Tamoxifen injection was performed at P10, and bones were collected after 3 weeks (at 5 weeks of age). Representative images of the proximal tibia (**A**) and the tibia diaphyseal area (**B**) Decreased fraction of Ai9^+^ cells migrated to the bone diaphysis in mutant animals compared with littermate controls (*n* = 3 per genotype; *n* = 2–3 fields counted per sample; *P = 0.03, by 2-tailed Student’s *t* test). Original magnification, ×20. (**C**) RNA-Seq of total femoral cDNA from *Rosa26*^mIfitm5/+^ *Acan-Cre* ERT2 mice (*n* = 3 per genotype) showed enrichment for chondrogenic gene expression in the mutant samples by GO term analysis. (**D**) Heatmap of focused differential gene expression analysis from RNA-Seq, showing upregulation of chondrogenic gene markers and decreased expression of osteogenic markers in the mutants compared with the control group.

The OI type V mouse models reported here recapitulated, at least in part, the human phenotype by exhibiting low bone mass, expansile cartilage formation, and growth restriction (9, 10). They had histologic and radiographic features similar to those reported in patients (8), including immature bone matrix with a mesh-like appearance (Figure 2B) and sclerotic bands in the femur metaphysis (Figure 5B). Additionally, *Rosa26^mIfitm5^* Prx1-Cre mice had joint deformities. To correlate this finding with the human phenotype, we performed a retrospective chart review of 7 individuals with OI type V from 3 skeletal centers in the United States and identified a variable degree of joint dysfunction in 6 of the 7 participants (85%, Supplemental Figure 11 and Supplemental Table 1). Taken together, our data suggest that mutant IFITM5 expression leads to abnormal chondrogenesis, while inhibiting osteo-chondroprogenitor cells’ maturation into bone. To support this hypothesis, we crossed the *Rosa*26*^mIfitm5^* *Acan-Cre ERT2* mice with Ai9 reporter mice [B6.Cg-*Gt(ROSA)26Sor^tm9(CAG-tdTomato)Hze^*/J], and traced reporter-positive cells 3 weeks after postnatal activation of the mutant *Ifitm5* allele. In *Rosa*26*^mIfimt5/Ai9^* *Acan-Cre ERT2* mice, cells coexpressing the Ai9 reporter and mutant *Ifitm5* showed altered distribution with retention in the growth plate area and decreased migration to the femur shaft (Figure 9, A and B), consistent with decreased differentiation into osteoblasts, whereas in littermate controls, *Acan-Cre ERT2^+^* cells marked with the Ai9 reporter showed the expected pattern of migration along the diaphysis (26). Next, we performed total RNA-Seq in *Rosa*26*^mIfitm5^* *Acan-Cre ERT2* mouse femurs to search for downstream transcriptomic changes in the long bones following activation of the mutant *Ifitm5* allele in chondrocytes. Gene ontology (GO) enrichment analysis in mutant femurs revealed enrichment for chondrocyte morphogenesis, proliferation, and differentiation processes (Figure 9C). A focused differential gene expression analysis revealed upregulation of chondrogenic markers and downnregulation of osteoblast markers (Figure 9D). Consistent with this finding, activation of the mutant *Ifitm5* allele in cultured marrow stromal cells (MSCs) via soluble Cre recombinase was associated with decreased osteoblastic differentiation and enhanced cell condensation toward chondrogenic differentiation in vitro (Supplemental Figure 12).

## Discussion

OI type V is one of the more common causes of moderate-to-severe OI, second only to collagen I–related forms (5–7). The pathogenic mechanism in OI type V is unknown, hindering the development of efficient and targeted therapies. We have previously generated a transgenic mouse model by overexpressing *Ifitm5* cDNA with the novel start codon, corresponding to the recurrent human OI type V mutation, under the control of the *Col1a1* 2.3 kb promoter (19). The mutant *Ifitm5* transgenic mice exhibited perinatal lethality and severe bone deformities, whereas the transgenic mice overexpressing wild type *Ifitm5* did not show significant abnormalities (19). Consistent with our transgenic mouse model, others subsequently published a CRISPR/Cas9-knockin mouse model that also exhibited a severe OI phenotype with perinatal lethality (20, 21), suggesting that a conditional, tissue- and stage-specific expression of the mutant *Ifitm5* may be an effective approach for mechanistic analyses. To our knowledge, we report here the first conditional mouse model for OI type V that expresses mutant IFITM5 in 3 different cell types, i.e., limb mesenchymal cells, osteogenic cells, and chondrogenic cell lineages. Mice expressing the mutant *Ifitm5* allele are viable and recapitulate the low bone mass and exuberant cartilage formation that is observed in humans with OI type V.

The phenotypic similarity and overlapping severity between the *Rosa26^mIfitm5^* Prx1-Cre and *Rosa26^mIfitm5^ Acan-Cre ERT2* mice support the notion that abnormal chondrogenesis is a major contributor to the skeletal phenotype. The expression of *IFITM5* in cartilage has been demonstrated in humans and mice (9, 15). Transcriptomics analysis has shown enrichment of *IFITM5* mRNA during chondrogenesis in human bone marrow mesenchymal cells (17) and in developing chick embryos (27). We confirmed this finding by showing that expression of IFITM5 was upregulated in conjunction with SOX9 in differentiating chondrogenic ATDC5 cells (Supplemental Figure 8). In *Rosa26^mIfitm5^* Prx1-Cre mice, expression of the mutant *Ifitm5* in mesenchymal progenitors of the osteo-chondrogenic lineage was associated with low bone mass and growth restriction, whereas the *Rosa26^mIfitm5^* Col1a1-Cre and *Rosa26^mIfitm5^* OC-Cre mice that expressed the mutant *Ifitm5* in committed or mature osteoblasts did not exhibit significant skeletal abnormalities (Figures 2 and 3, and Supplemental Figures 3 and 4).

Previously published OI type V mouse models demonstrated a severe reduction in osteoblast differentiation and replacement of the normal bone tissue with cartilaginous matrix (19, 20). It is possible that the expression of mutant IFITM5 in skeletal progenitor cells alters their lineage differentiation in early development. Consistent with this hypothesis, we found that the percentage of periosteal skeletal progenitor cells was significantly increased in the mutant *Rosa26^mIfitm5^* Prx1-Cre mice (Figure 4, A and B). Moreover, histological sections in 2-month-old *Rosa26^mIfitm5^* Prx1-Cre mice showed the failure of chondrogenic terminal differentiation and impaired endochondral ossification (Figure 5C). In the *Rosa26^mIfitm5^ Acan-Cre ERT2–*mutant mice that had a skeletal phenotype that overlapped with the *Rosa26^mIfitm5^* Prx1-Cre model, tracing of cells coexpressing the Ai9 reporter and mutant *Ifitm5* showed decreased differentiation into osteoblasts, and the bone transcriptome showed enrichment for chondrogenic marker expression (Figure 9). These data suggest that mutant IFITM5 could maintain or induce osteo-chondro progenitor cell numbers while inhibiting their terminal differentiation. This then could reduce the pool of osteoblastic cells derived from progenitors in the growth plate (28).

The biochemical mechanisms whereby mutated IFITM5 protein causes the skeletal pathology in OI type V are not well understood. Unlike other forms of OI that are associated with abnormalities in type I collagen (i.e., qualitative or quantitative defects of type I collagen, and abnormal processing of type I collagen), our previous transgenic mouse model failed to reveal any significant change in the distribution of collagen throughout the bone or in its posttranslational modifications (19). Since IFITM5 forms part of a protein complex at the membrane (29) and the localization of the mutant IFITM5 is not altered (30), it is possible that a neomorphic effect on protein-protein interactions at the plasma membrane may alter downstream signaling cascades. Our proteomics analysis using RPPA identified increased levels of pERK1/2 in the calvaria of transgenic *Ifitm5*^c.-14C>T^ mice. Further studies in cells and zebrafish overexpressing mutant IFITM5 support our observation that activation of this pathway is involved in the mineralization defect and abnormal cartilage development in OI type V (Figure 6 and Supplemental Figure 7). The ERK/MAPK signaling pathway has a critical role in both cartilage and bone development. Previous studies have shown that this pathway may regulate osteoblast differentiation by activating downstream RUNX2 and OSX-mediated transcription of osteogenic genes (31). ERK signaling has been implicated in pigment epithelium–derived factor–mediated (PEDF-mediated) regulation of osteoblast maturation and mineralization (32). Inactivation of ERK1 and ERK2 in mesenchymal osteo-chondroprogenitor cells in *ERK1*^–/–^ *ERK2^fl/fl^* Prx1-Cre mice disrupted osteoblast differentiation and resulted in severe bone deformities (33). In agreement with these findings, activation of MAPK signaling via transgenic overexpression of a constitutively active MEK1 stimulated osteoblast differentiation and mineralization (34). Conversely, somatic mosaicism for gain-of-function mutations in *MAPK2* in melorheostosis was associated with increased osteoid formation and decreased mineralization in vivo and impaired osteoblast differentiation in vitro (35). Activation of ERK signaling was also implicated in the mechanism of a rare form of OI due to loss-of-function variants in *CCDC134* (36). These seemingly conflicting effects suggest a cell- and stage-specific action of this pathway, and likely a crosstalk with other signaling events during osteoblast differentiation (37, 38).

Coactivation of ERK signaling and SOX9 expression has been reported in differentiating osteoblasts (39) and in developing cartilage (40), and they are both important regulators of skeletal development throughout osteo-chondral differentiation. SOX9 is regulated by the ERK/MAPK signaling pathway (39, 41, 42). We observed altered spatiotemporal expression of *Sox9* and increased SOX9 protein levels in our mouse models. Moreover, genetic deletion of *Sox9* in cells expressing mutant IFITM5 led to improvement in the growth plate architecture and improved the low bone mass phenotype (Figure 8), supporting the hypothesis that SOX9 plays a critical role in the pathological mechanism of OI type V. SOX9 is a master transcription factor in chondrocyte differentiation (43, 44) and is downregulated in hypertrophic chondrocytes. Persistent expression of *Sox9* in the hypertrophic zone disrupts terminal chondrocyte maturation and endochondral bone formation (45–47). Interestingly, impaired chondrocyte hypertrophy, as seen in *Rosa26^mIfitm5^* Prx1-Cre–mutant mice (Figure 3, C and D), has been reported in mouse models that have constitutively activated FGFR3 and MAPK signaling (48–52). In mice expressing the thanatophoric dysplasia type II activating *FGFR3* mutation, the defect in chondrocyte maturation and bone formation was linked to increased SOX9 protein stability (51, 52). Moreover, *Sox9* has been shown to downregulate *Runx2* (53), or inhibit its activity (54), during both chondrocyte hypertrophy and endochondral ossification. In humans, campomelic dysplasia caused by *SOX9* haploinsufficiency is associated with chondrocyte hypertrophy and increased RUNX2 levels (44, 54). Hence, gain of SOX9 function can affect not only chondrogenesis, but also bone formation derived from chondrogenic cells in the growth plate.

Conditional expression of mutant *Ifitm5* in the *Rosa26^mIfitm5^* Prx1-Cre and *Rosa26^mIfitm5^ Acan-Cre ERT2* mice resulted in a low bone mass phenotype, as seen in individuals with OI type V. Biomechanical testing in the *Rosa26^mIfitm5^ Acan-Cre ERT2* model demonstrated reduced ductility and increased bone stiffness, correlating with the findings in other OI mouse models (55, 56). Although not associated with rhizomelic short stature, individuals with OI type V can present with variable longitudinal growth restriction (9, 10), and clinical observations have led others to suggest a defect in growth plate development and function (10, 57). Our mutant mice exhibited cartilage overgrowth at the knees and ankles that progressed to joint contractures (Figure 5). Interestingly, severe joint contractures and ankylosis have been reported in some individuals with OI type V (58). In a small cohort of patients, we observed milder joint dysfunction, most frequently manifested as limited range of motion at the hips and elbows (Supplemental Figure 11 and Supplemental Table 1).

A limitation of our model is the expression of the mutant protein from the *Rosa* locus. The Prx1-Cre mouse model targets cells of the limb bud and cranial mesenchyme, while sparing the axial skeleton, and thus was not suitable to model scoliosis or other spinal abnormalities. Our mouse models demonstrated abnormal cartilage formation, but they did not recapitulate the hypertrophic callus or calcification of interosseous membranes as seen in the human patients. However, the similar tissue abnormalities in the human studies both here and in the literature, the findings in heterologous systems in zebrafish and cell models (MC3T3), and the rescue of both cell and fish phenotypes with ERK inhibition and of the mouse phenotype by genetic deficiency of *Sox9*, together support the relevance of these mechanistic findings to the human condition.

In summary, we present here a conditional mouse model for OI type V that expresses mutant *Ifitm5* allele under the *Rosa26* promoter. Conditional expression of mutant *Ifitm5* in cells of the osteo-chondroprogenitor or chondrogenic lineages resulted in low bone mass, growth restriction, cartilage overgrowth, and joint deformities mimicking the human phenotype. Importantly, we demonstrated a chondrogenesis defect that, together with a mineralization defect, is likely driven by activated ERK signaling and elevated SOX9 downstream of the *IFITM5* mutation. The rescue by ERK inhibition and *Sox9* deficiency points to potential therapeutic approaches for this disorder and have a broader impact on the understanding of mechanisms governing skeletal homeostasis.

## Methods

### Sex as a biological variable.

Both male and female mice were used for the experiments described here. For zebrafish study, sex-specific differences were not considered because zebrafish sex differentiation occurs at 21–23 dpf, whereas all experiments reported here were concluded by 5 dpf. The human study included both male and female participants.

### Ifitm5 conditional Rosa26-knockin mouse model.

We generated a knockin *Rosa26* mouse model by introducing the mutant *Ifitm5* cDNA (m*Ifitm5*) into the *Rosa26* locus using previously described approaches (22, 59). The murine *Ifitm5* cDNA was introduced into the pCMV-Tag1 vector to add an in-frame Flag tag at the C-terminus, and in vitro mutagenesis was performed to obtain the c.-14C>T mutation. The Flag-tagged mutant cDNA was then introduced into the pRosa26-DEST vector using Gateway recombination cloning. pRosa26-DEST was a gift from Nick Hastie and Peter Hohenstein (Addgene plasmid no. 21189; http://n2t.net/addgene:21189; RRID:Addgene_21189; ref. 59). Targeting of the construct to the *Rosa26* locus in C57BL/6N mouse embryonic stem (ES) cells (JM8A3) was performed via electroporation by the Genetically Engineered Rodent Model core at Baylor College of Medicine (BCM). ES cell clones were screened for correct targeting by PCR amplification of the 5′ region, the knocked-in Ifitm5 cDNA insert, and the 3′ region within the *Rosa26* locus (Supplemental Table 2). Correct copy number (heterozygosity) of the knocked-in allele was validated by RT-qPCR using a Neo TaqMan gene dosage assay. Following initial PCR screening, selected clones were validated by Southern blotting, as previously described (22). Correctly targeted ES cells were then expanded and assessed for morphology and karyotyping prior to the generation of chimeric animals via microinjection and transfer of C57BL/6NTac-A^tm1.1Arte^ Tyr^tm1Arte^ (Taconic model number 11227) blastocysts. Sanger sequencing confirmed the insertion of the knocked-in allele in founder animals. Chimeras were crossed with C57BL/6NTac-A^tm1.1Arte^ Tyr^tm1Arte^ mice to generate N1 animals, inheritance of the knockin allele in N1 animals was confirmed by Sanger sequencing, and the N1 generation was backcrossed with C57BL/6 mice to generate N2 mice. For all studies described here, mice were maintained on a C57BL/6 genetic background. Mice that were heterozygous for the *Rosa26*-knockin mutant *Ifitm5* allele (*Rosa26*^mIfitm5/+^) were crossed with expressing *Cre/–* to generate the following genotypes: mutant (*Rosa26^mIfitm5/+^ Cre*/– mice) and littermate controls (including *Cre*/– mice, *Rosa26*^mIfitm5/+^ mice, and *Rosa26*^+/+^ mice). The Cre mouse lines that were used include the Prx1-Cre transgenic line, the OC-Cre transgenic line, the rat 2.3 kb Col1a1 transgenic line Tg(Col1a1-Cre)2Bek (Col1a1-Cre), and the *Agc1^tm(IRES-CreERT2)^* line (*Acan-Cre* ERT2) that expresses tamoxifen-inducible CreER^T2^ from the aggrecan promoter. The *Acan-Cre* ERT2–inducible Cre recombinase was activated by administration of tamoxifen (10 mg/kg/dose) via i.p. injections, starting at P10 and continuing until P15. For *Sox9^fl^* rescue experiments, *Rosa*26*^mIfitm5^* *Acan-Cre ERT2* mice were crossed with *Sox9^fl/fl^* mice, Cre recombinase was activated as above, and the following littermates were collected for analysis: *Rosa*26*^mIfitm5^* *Sox9^fl/+^* *Acan-Cre ERT2* and *Sox9^fl/+^ Acan-Cre ERT2* (compared with *Rosa26^mIfitm5^ Acan-Cre ERT2*). In addition, *Rosa*26*^mIfitm5^* *Acan-Cre ERT2* mice were crossed with the *Rosa26*-knockin tdTomato reporter line [B6.Cg-*Gt(ROSA)26Sor^tm9(CAG-tdTomato)Hze^*/J, herein referred to as Ai9 reporter mice] to generate *Rosa*26*^mIfimt5/Ai9^* *Acan-Cre ERT2* mice.

### Generation of IFITM5-expressing zebrafish.

The expression of mutant IFITM5 in zebrafish (*Danio rerio*) was achieved by injecting zebrafish embryos (*AB* strain) with mutant human *IFITM5* mRNA at the 1-cell stage (30 minutes to 1 hpf). In brief, human wild type or mutant *IFITM5* cDNA was introduced into the pCS2^+^ expression vector. To obtain the human *IFITM5* cDNA, total mRNA was extracted from induced pluripotent stem cells (iPSCs) that were generated from an individual with OI type V and differentiated into osteoblasts for 14 days using previously described methods (60, 61). Total mRNA served as a template to generate cDNA (Superscript III First Strand RT-PCR Kit, Invitrogen, Thermo Fisher Scientific), and mutated or wild type *IFITM5* cDNAs were amplified (Supplemental Table 2). Mutated and wild type *IFITM5* mRNA or *mCherry* mRNA was transcribed in vitro from the pCS2^+^ vector using the mMessage SP6 Transcription Kit (Invitrogen, Thermo Fisher Scientific), and 100 pg/embryo was injected. At 120 hpf, embryos were fixed in 4% paraformaldehyde (PFA) and then stained in 0.1% Alcian blue solution. For ERK/MEK inhibitor rescue experiments, embryos were injected with mutant *IFITM5* mRNA and then randomly allocated at 5 hpf to treatment (InSolution MEKI/II Inhibitor III, PD0325901, EMD Millipore at 0.5 μM) or control (DMSO) groups. Treatment was applied daily to fresh E3 water, and at 120 hpf embryos were fixed and stained as described above for morphological assessment.

### Cell culture.

All cells were cultured at 37°C and 5% CO_2_ in a humid environment. MC3T3-E1 or ATDC5 cells (American Type Culture Collection [ATCC]) were cultured according to ATCC guidelines. A Tet-On system was used for stable expression of *Ifitm5^c.-14C>T^* under doxycycline induction in MC3T3 cells. In brief, cells were transfected with a PiggyBac-based system to insert 2 transgenes into the genome: *Ifitm5^c.-14C>T^* (pTight-TRE- *Ifitm5^c.-14C>T^*) and rtTA (tetracycline-responsive transactivator, pCMV-rtTA). Transfected cells were selected with 2 μg/mL puromycin (Gibco, Thermo Fisher Scientific) and 500 μg/mL G418 (Gibco, Thermo Fisher Scientific). Stable cells were maintained in growth media (αMEM with 10% FBS, 1% glutamine, and 1% penicillin/streptomycin) containing puromycin and G418. *Ifitm5^c.-14C>T^* expression was induced by doxycycline administration (1.2 μM, MilliporeSigma). For in vitro osteogenic differentiation, cells were incubated for 14 days in growth media (αMEM with 10% FBS, 1% glutamine, and 1% penicillin/streptomycin) containing β glycerophosphate (10 mM, MilliporeSigma), ascorbic acid (100 μg/mL, MilliporeSigma), and BMP2 (100 ng/mL, R&D Systems). ERK inhibitor rescue was studied by applying U0126 (MilliporeSigma, at 5 or 10 μM) or vehicle (methanol). Alizarin red S staining was performed after fixation of cells with 4% formalin. For in vitro chondrogenic differentiation of ATDC5, cells were incubated for 21 days in growth media (DMEM/F12 with 10% FBS, 1% glutamine, and 1% penicillin/streptomycin), containing insulin-transferrin-selenium (ITS) supplement (Gibco, Thermo Fisher Scientific).

### MSC differentiation in vitro.

Flushed bone marrow cells from *Rosa26*-knockin mutant *Ifitm5* mice (*Rosa26*^mIfitm5/+^) were treated with 2 μM TAT Cre recombinase (Excellgen, EG-1001) in low-serum growth media (αMEM with 1% FBS, 1% glutamine, and 1% penicillin/streptomycin) to activate the *Ifitm5* mutation. For osteoblast differentiation, MSCs were plated at 8 × 10^5^/mL into a 12-well plate, incubated with Cre recombinase overnight, and allowed to recover for 24 hours in serum-rich growth media (20% FBS). Osteogenic differentiation media (αMEM with 10% FBS, 1% glutamine, 1% penicillin/streptomycin, 10 mM β glycerophosphate, and 100 μg/mL ascorbic acid) were applied to confluent cultures and changed every other day for a total of 7 days before RNA was extracted for RT-qPCR analysis. For chondrogenic differentiation, MSCs were pretreated with Cre recombinase and then plated at 1.5 × 10^6^/mL (150,000 cells/well) into v-shaped 96-well plates and spun down (500*g*, 5 min) to pellet culture. Chondrogenic media (StemXVivo Chondrogenic Media and Chondrogenic Supplement, R&D Systems) were refreshed every 3 days for a total of 21 days. Cells were fixed in 4% glutaraldehyde and stained with Alcian Blue. Condensed cartilaginous structures were embedded in OCT media (Tissue-Tek O.C.T. Compound, Sakura) and cryosectioned at 7 μm for staining.

### Isolation and flow cytometric analysis of periosteal and bone marrow skeletal progenitor cells.

To isolate periosteal cells, dissected femurs and tibia were placed in PBS, and the overlaying muscles were carefully removed. Bones were then incubated in ice-cold 1% FBS for 15 minutes, and the loosely associated periosteum was scraped off using forceps. The periosteal tissues were incubated with 0.2% collagenase and 10% FBS in PBS at 37°C for 1 hour. The dissociated periosteal cells were washed in PBS, filtered with a 40 μm cell strainer, and resuspended at approximately 1 × 10^7^ cells/mL. To isolate bone marrow cells, femurs and tibia cleaned of periosteum were crushed in 1% FBS using a mortar and pestle. The remaining bone was further fragmented using dissection scissors. The bone fragments in 1% FBS were washed and then incubated in 0.2% collagenase and 10% FBS in PBS at 37°C for 1 hour, as previously described (62). After incubation the dissociated cells were washed in PBS, filtered through a 40 μm cell strainer, and resuspended at approximately 1 × 10^7^ cells/mL. To analyze skeletal stem/progenitor cell populations, cells were stained with CD45 Pacific Blue (clone: 30-F11), CD31-eFluor 450 (clone: 390), and Ter119-BV605 (clone: Ter119) in combination with CD140a-APC (clone: APA5) and CD105-PE-Cy7 (clone: MJ7/18). Antibodies were purchased from eBioscience. DAPI was used for viable cell gating. Flow cytometric experiments were performed at the BCM Cytometry and Cell Sorting Core using an LSR Fortessa (BD Biosciences). Data were analyzed with FlowJo Software (TreeStar) and are represented as dot plots of fluorescence intensity.

### RNA extraction, RT-qPCR, and RNA-Seq.

Bone tissues (femur or tibia) were collected from 2-month-old *Rosa26^mIfitm5/+^* Prx1-Cre, *Rosa26^mIfitm5/+^* OC-Cre and *Rosa26^mIfitm5/+^* Col1a1-Cre mice. Cartilage tissue (rib cage) was collected at P6 from *Rosa26^mIfitm5/+^ Acan-Cre* ERT2 mice that had been treated at P3–P5 with s.c. injection of tamoxifen (10 mg/kg/dose). Total RNA was extracted in TRIzol Reagent (Ambion) using TissueLyser (QIAGEN). The iScript cDNA synthesis kit (Bio-Rad) or SuperScript III (Invitrogen, Thermo Fisher Scientific) was used to synthesize cDNA according to the manufacturer’s protocol. RT-qPCR was performed on a LightCycler instrument (Roche) using FastStart Essential DNA Master Reagent (Roche) with *GAPDH* or *B2m* (β-2 microglobulin) as an internal control. For RNA-Seq, femurs were collected from 5-week-old *Rosa26^mIfitm5/+^ Acan-Cre* ERT2 mice that were treated at P10 with i.p. injection of tamoxifen (10 mg/kg/dose). Total RNA from pulverized bone was extracted using TRIzol Reagent (Ambion) and further purified by phenol-chloroform precipitation. Total RNA (1 μg) was subjected to rRNA depletion–based library preparation and sequencing (Azenta Life Sciences). Between 25.5 and 34 million paired-end reads were generated for each sample. The alignment was performed using STAR aligner with mm39 as the reference genome. Normalization, differential expression, hierarchical clustering, and GO analysis were then performed using the RNA-Seq analysis pipeline in Partek Genomics Suite (Partek). Differential gene expression analysis was performed using DESeq2, and statistical significance was determined by 2-way ANOVA, which is built into the Partek Genomics Suite RNA-Seq analysis pipeline. The significantly differentially expressed genes (fold change >2 and FDR <0.05) were then used for GO analysis. Chondrogenic and osteogenic genes were selected for heatmap generation on the basis of a previous report (63).

### Protein extraction, RPPA, and Western blotting.

Protein was extracted from calvaria of transgenic *Ifitm5*^c.-14C>T^ mice (19) in modified Tissue Protein Extraction Reagent (Thermo Fisher Scientific) containing a protease and phosphatase inhibitor cocktail (Roche), using TissueLyser (QIAGEN). The protein concentration was measured using a BCA protein assay kit (Thermo Fisher Scientific), and the lysate was diluted to 0.5 mg/mL in RPPA lysis buffer containing SDS sample buffer and 0.25% β-mercaptoethanol. The RPPA was conducted at the BCM Antibody-based Proteomics Core using a standardized protocol (64). In total, 8 mutant transgenic (*Ifitm5^c.-14C>T^) mice,* 8 wild type transgenic mice, and 8 nontransgenic littermate bone samples were included in the experiment. Each sample was assayed in 3 technical replicates to account for technical variation. For Western blot analysis, 25–50 μg total protein was loaded onto a 4%–15% SDS-PAGE gradient gel (Bio-Rad) and transferred onto a PVDF membrane (MilliporeSigma). Membrane was blocked in 5% skim milk and incubated overnight at 4°C with a primary antibody: anti–p–p44/42 MAPK (ERK1/2) Thr202/Tyr204 (Cell Signaling Technology, 9101S),anti–p44/42 MAPK (ERK1/2) (Cell Signaling Technology, 4695S), anti-SOX9 (EMD Millipore, AB5535), anti-IFITM5 (Abcam, ab230863), anti–α-tubulin (MilliporeSigma, T5168), and anti-GAPDH (MilliporeSigma, G9295). Signal was captured by film or via ChemiDoc imager (Bio-Rad).

### Histology and IHC.

Bone samples from a patient with OI type V and an unaffected control were obtained when the participants were undergoing skeletal surgery for a medical indication. The fragments of bone removed during surgery, which otherwise would have been discarded, were collected and processed for histological analysis. Mouse hind limbs were collected from mice at 2 weeks and 2 months of age. Tissues were fixed in 4% PFA overnight at 4°C and stored in 70% ethanol. Paraffin-embedded tissues were sagittally sectioned at 7μm. H&E staining, Alcian Blue staining, and Nuclear Fast Red staining (MilliporeSigma) were performed according to standard methods. Toluidine blue staining was performed using the manufacturer’s instructions (VitroView). For IHC, samples were deparaffinized, refixed in 4% PFA for 30 minutes, and incubated at 60°C in Target Retrieval Solution, pH 6.1 (Dako) for antigen retrieval. Samples were treated in 3% hydrogen peroxide for 30 minutes at room temperature, followed by blocking with 5% normal goat serum. The following primary antibodies were applied overnight at 4°C: anti-COL10A1 (DSHB, X-AC9), anti-SOX9 (EMD Millipore, AB5535), or anti–p–p44/42 MAPK (ERK1/2) Thr202/Tyr204 (Cell Signaling Technology, 9101S). Anti-rabbit secondary antibody (Vectastain ABC System, Vector Laboratories) was applied, and DAB (Vector SK-4100) staining was performed per the manufacturer’s instruction. Slides were stained with hematoxylin and mounted using Cytoseal XYL xylene-based mounting medium (Thermo Fisher Scientific). Images were taken with a light microscope (Axioplan 2, Zeiss) or with a Zeiss AxioScan_Z1 whole slide scanner by the BCM RNA In-Situ Hybridization core.

### Skeletal radiographs.

Radiographic images of *Rosa26^mIfitm5/+^ Cre*/– mice were taken with the Kubtec XPERT80 (Kubtec X-ray).

### Skeletal preparations.

P21 *Rosa26^mIfitm5/+^ Cre*/– mutant mice and littermate controls were sacrificed and fixed with 95% ethanol, stained overnight with Alcian blue (containing 0.015% Alcian blue 8GX, 20% acetic acid, and 80% ethanol 95%), and then soaked in 2% KOH solution for 24 hours to remove remnants of soft tissue. Skeletal preparations were then stained overnight in alizarin red solution (containing 0.005% alizarin red S in 1% KOH), cleared in 1% KOH/20% glycerol solution, and stored in glycerol/95% ethanol 1:1 solution.

### Immunofluorescence imaging.

Bones were collected from 5-week-old *Rosa*26*^mIfimt5/Ai9^* *Acan-Cre ERT2* mice, fixed in 4% PFA, and decalcified in 10% ETDA. Samples were embedded in OCT media (Tissue-Tek O.C.T. Compound, Sakura), cryosectioned at 10 μm, and mounted onto adhesive microscope slides using the Tape-Transfer system (Leica). Slides were counterstained with DAPI (IHC World) and sealed using ProLong Glass Antifade mounting media (Invitrogen, Thermo Fisher Scientific) according to the manufacturer’s instructions. Images were acquired with a Zeiss LSM 780 confocal microscope at the BCM Optical Imaging and Vital Microscopy Core. Images were processed with ImageJ software (NIH).

### Micro CT analysis.

Spines and right femurs were scanned in 70% ethanol using a Scanco μCT-40 micro CT system (Scanco Medical, 55kVp and 145 μA X-ray source), and scans were reconstructed at a 16 μm isotropic voxel size. Trabecular bone of L4 vertebrae and the distal metaphyses of right femurs were analyzed using Scanco software by manually contouring trabecular bone. For vertebrae, the region of interest (ROI) was defined as the trabecular volume between the L4 vertebral endplates. For femurs, the trabecular ROI was defined proximal to the distal femoral growth plate, and the number of slides analyzed was adjusted to the femur length (femur length was measured from the top of the femoral head to the bottom of the medial condyle): 75 slides (1.2 mm) were analyzed for 2-month-old Prx1-Cre and OC-Cre mouse model control femurs; 55–60 slides were analyzed for 5-week-old *Acan-Cre ERT2* control femurs; and 45–50 slides were analyzed for mutant mouse femurs (across all models). Quantification of trabecular parameters was performed using Scanco software with a threshold value of 230. These parameters included BV/TV, trabecular number (Tb.N), trabecular thickness (Tb.Th), connectivity density (Conn.D), and tissue mineral density (TMD) (65). Cortical bone parameters of the femoral midshaft were measured at the exact center and at the distal 75% of femur length using the automated thresholding algorithm included in the Scanco software. Trabeculae in contact with cortical bone were manually removed from the ROI (11 slides analyzed per location; threshold of 210). The cortical parameters included the total cross-sectional area (Tt.Ar), the cortical bone area (Ct.Ar), the marrow area (Ma.Ar), cortical thickness (Ct.Th), cross-sectional moments of inertia (CSMI), the anterior-posterior diameter, and the TMD (65). Six to 8 spines and femurs were scanned per group.

### Biomechanical measurement.

Femurs were collected from 5-week-old *Rosa26^mIfitm5/+^ Acan-Cre* ERT2 mice that were treated with i.p. injection of tamoxifen (10 mg/kg/dose) at P10. Bones were collected, stripped of soft tissues, wrapped in saline-soaked gauze, and frozen at –20°C until analyses were performed. The femurs were tested to failure in 3-point bending at a rate of 0.1 mm/sec and were oriented in the test fixture such that the anterior surface was in compression and the posterior surface in tension. Femurs were tested wet at room temperature using the displacement mode with an Instron 68SC-2 microtester (Instron Inc.). The test fixture span was 3.61 mm. A 100N load cell was used to collect data, and the load and displacement data were captured at a rate of 40 Hz by using BLUEHILL Software (Instron 68SC-2). The maximum load was determined by finding the highest load value recorded before the specimen fractured. The region of the load displacement curve between 1N and the maximum load was separated into 5 segments, and the fitted line of the segment with the greatest slope was defined as the stiffness. A line representing 10% degradation of this stiffness was used to define the yield point. The elastic region was identified as the region from the completion of the preload to the yield point. The post-yield region was identified as the region from the yield point until the point of specimen fracture. Using a trapezoidal numerical integration method, the work to fracture was calculated as the area under the load displacement curve. The cross-sectional geometry of each bone as determined by micro CT image analysis was used to convert the maximum load and stiffness data into ultimate stress and elastic modulus values using beam theory.

### Chart review of patients with OI type V.

We conducted a retrospective chart review for 7 individuals with OI type V at 3 expert skeletal centers in the United States: BCM (Houston, Texas), Oregon Health and Sciences University (Portland, Oregon), and Omaha Children’s Hospital (Omaha, Nebraska). The following data were collected from the chart review: age, sex, molecular diagnosis of OI type V, the presence or absence of typical OI type V clinical features (hyperplastic callus, calcification of the interosseous membrane, radial head dislocation), and the presence or absence of joint pathology (decreased range of motion, joint contracture, or joint ossification in the shoulders, elbows, wrists, hips, knees, and ankles). The data were collected in a standardized format using specific data fields from all 3 centers.

### Statistics.

For all statistical analyses, parametric unpaired tests were used, including 1-way ANOVA and 2-tailed Student’s *t* test. Prism GraphPad (GraphPad Software) or SAS 9.4 (SAS Institute Inc., 2007) was used for statistical analysis. *P* values of less than 0.05 were considered significant. Data are presented as the mean ± SD.

### Study approval.

Mice and zebrafish were housed in the BCM Animal Vivarium, and all procedures were approved by the IACUC of BCM. Bone samples from an individual with OI type V and an unaffected individual were obtained when these individuals were undergoing skeletal surgery for a medical indication. The fragments of bone removed during surgery, which otherwise would have been discarded, were collected and processed under a protocol approved by the IRB of BCM. Informed consent was obtained from the parents or legal guardians prior to collection of samples. For the retrospective chart review, medical records for 6 individuals were reviewed after informed consent was obtained from the individuals or their legal guardians. These 6 individuals were enrolled in an observational natural history study of OI being conducted by the NIH Rare Diseases Clinical Research Network’s Brittle Bone Disorders Consortium (BBDC) (NCT02432625). For 1 participant, who was not enrolled in the observational study, written permission was obtained to review and publish deidentified phenotypic data.

### Data availability.

All data generated or analyzed in this study are included in the manuscript and supporting files. A Supporting Data Values file has been provided for all numerical data. RNA-Seq data were deposited in the NCBI’s Gene Expression Omnibus (GEO) database (GEO GSE268601).

## Author contributions

RM, BHL conceptualized the study. Methodology and investigation: RM, IWS, MEW, ASB, VCR, LO, YJ, CDL, DB, ECB, MMJ, YC, RC, IL, MA, CL, DNM, CGA, DP, and JDH designed the study methodology and performed experiments. BCD, AC, BMS, MJ, and SH acquired data and performed statistical analyses. AAT, AT, MW, EO, VS, and SCSN recruited study participants and conducted clinical phenotyping. BHL supervised the work. Writing – original draft: RM and BHL wrote the original draft of the manuscript. All authors reviewed and edited the manuscript.

## Supplementary Material

Supplemental data

Unedited blot and gel images

Supporting data values

## Version 1 06/17/2024

In-Press Preview

## Version 2 08/01/2024

Electronic publication
