# Supplementary material for: The IFITM5 mutation in osteogenesis imperfecta type V is associated with an ERK/SOX9-dependent osteoprogenitor differentiation defect
Source: J Clin Invest. 2024 Jun 17;134(15):e170369. doi: 10.1172/JCI170369 (PMC11290974; doi:10.1172/JCI170369)
Supplement: Supplemental data [file jci-134-170369-s259.pdf]

The *IFITM5* mutation in osteogenesis imperfecta type V is associated with an ERK/SOX9-dependent osteoprogenitor differentiation defect

Supplemental materials:

Fig. S1 to Fig. S12

Table S1, S2

Figure S1

A

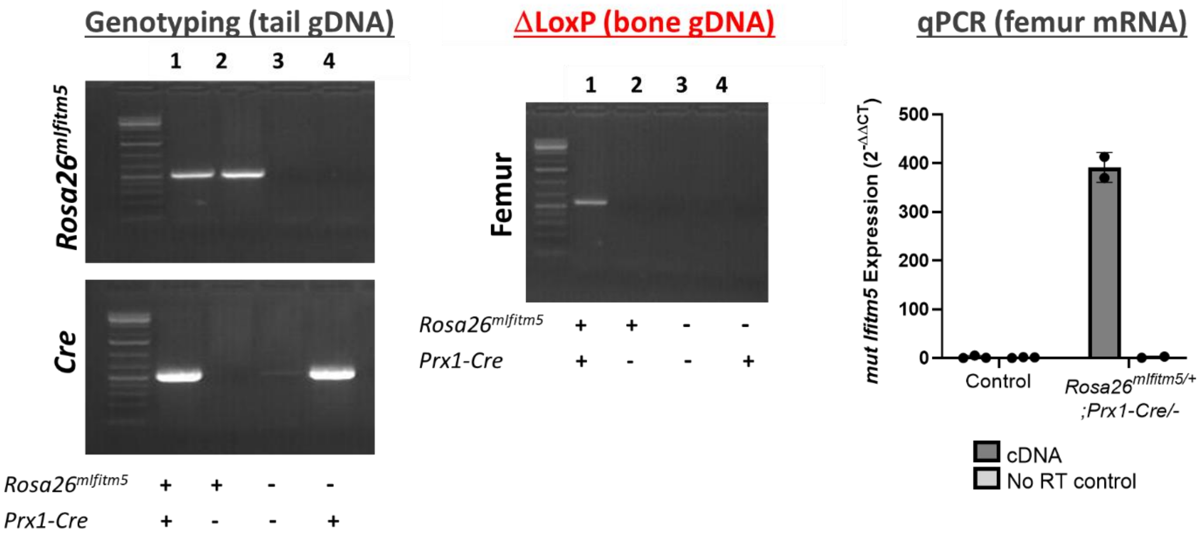
Genotyping (tail gDNA)
1 2 3 4
Rosa26mIfitm5
Cre
Rosa26mIfitm5 + + - -
Prx1-Cre + - - +
ΔLoxP (bone gDNA)
1 2 3 4
Femur
Rosa26mIfitm5 + + - -
Prx1-Cre + - - +
qPCR (femur mRNA)
mut Ifitm5 Expression (2-ΔΔCT)
500
400
300
200
100
0
Control
Rosa26mIfitm5/+ ;Prx1-Cre/-
cDNA
No RT control

B

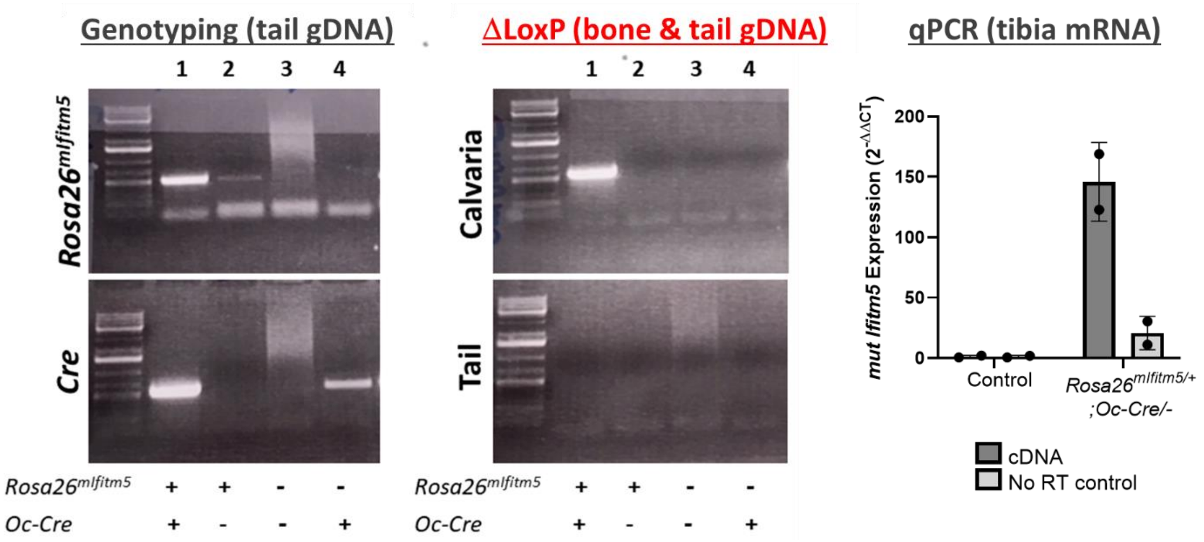
Genotyping (tail gDNA)
1 2 3 4
Rosa26mIfitm5
Cre
Rosa26mIfitm5 + + - -
Oc-Cre + - - +
ΔLoxP (bone & tail gDNA)
1 2 3 4
Calvaria
Tail
Rosa26mIfitm5 + + - -
Oc-Cre + - - +
qPCR (tibia mRNA)
mut Ifitm5 Expression (2-ΔΔCT)
200
150
100
50
0
Control
Rosa26mIfitm5/+ ;Oc-Cre/-
cDNA
No RT control

C

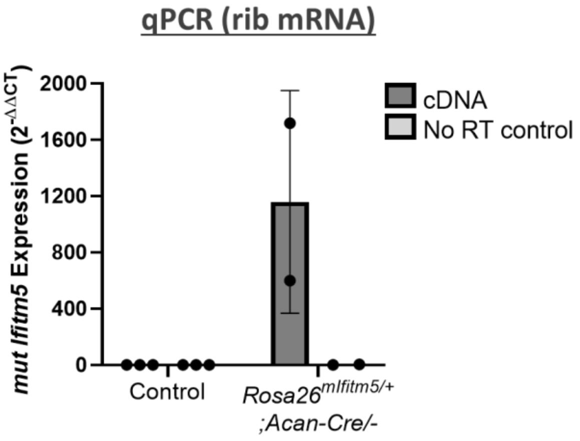

D

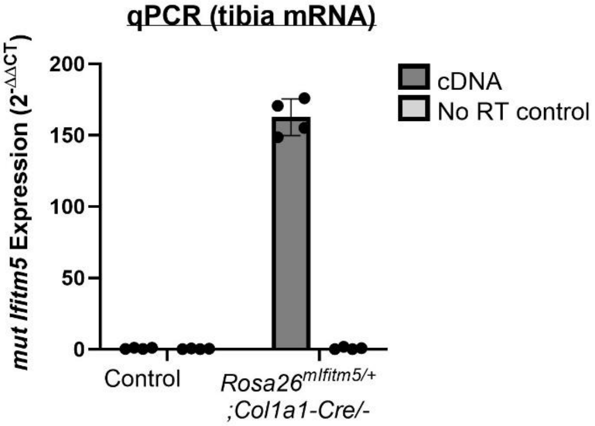

Fig. S1: Conditional expression of the mutant *Ifitm5* allele in *Rosa26$^{mIfitm5/+}$;Cre/-* mice. (A) *Rosa26$^{mIfitm5/+}$*;Prx1-Cre mouse model. Representative gel image of PCR amplification validating the deletion of LoxP cassette (DLoxP), and quantitative real-time (qPCR) analysis showing the expression of mutant *Ifitm5* cDNA in femurs. (B) *Rosa26$^{mIfitm5/+}$*;OC-Cre mouse model. Representative gel image of PCR amplification validating the deletion of LoxP cassette (DLoxP) in calvaria, and qPCR analysis showing the expression of mutant *Ifitm5* cDNA in tibia. (C) *Rosa26$^{mIfitm5/+}$*;*Acan-Cre* ERT2 mouse model. qPCR analysis showing the expression of mutant *Ifitm5* cDNA in rib cartilage. (D) *Rosa26$^{mIfitm5/+}$*;Col1a1-Cre mouse model. qPCR analysis showing the activation of mutant *Ifitm5* expression in tibia.

Figure S2

A

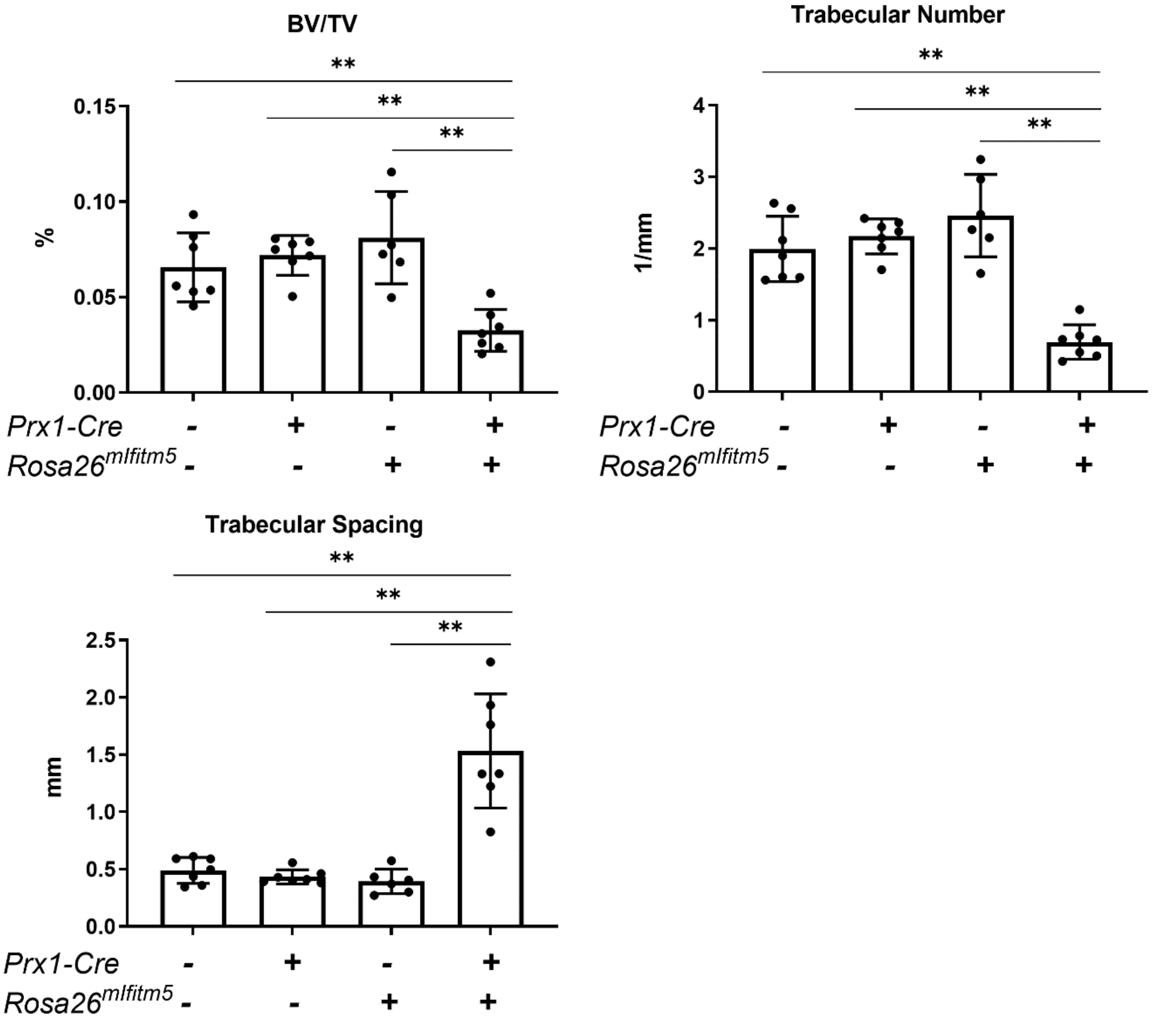
BV/TV
**
**
**
0.15
0.10
0.05
0.00
%
Prx1-Cre - + - +
Rosa26mIfitm5 - - + +
Trabecular Number
**
**
**
4
3
2
1
0
1/mm
Prx1-Cre - + - +
Rosa26mIfitm5 - - + +
Trabecular Spacing
**
**
**
2.5
2.0
1.5
1.0
0.5
0.0
mm
Prx1-Cre - + - +
Rosa26mIfitm5 - - + +

B

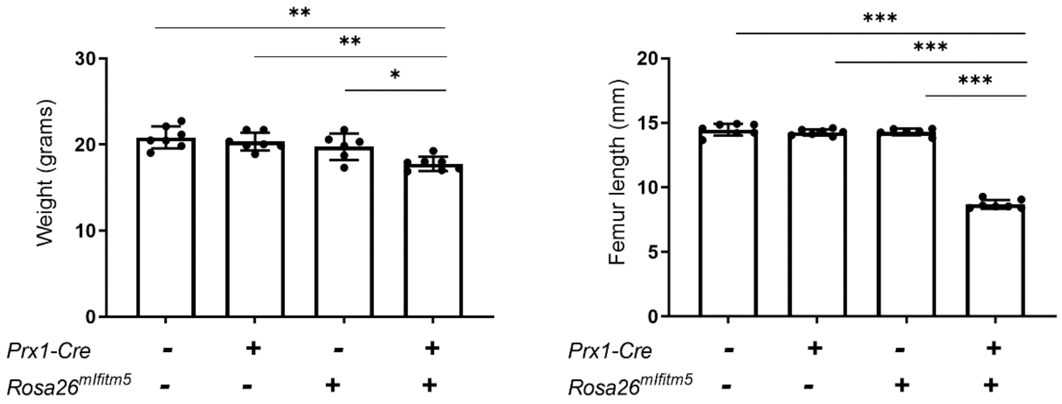

Fig. S2: Conditional expression of the mutant *Ifitm5* allele results in low bone mass and growth retardation in *Rosa26*$^{mIfitm5/+}$;Prx1-Cre female mice. (A) Micro CT analysis in femurs showed significant decrease in bone architectural parameters in *Rosa26*$^{mIfitm5/+}$;Prx1-Cre mutants, including the bone volume/total volume (BV/TV), and trabecular number. Consistently, trabecular spacing was increased. Analysis preformed in 2 months old female (One-way ANOVA with Tukey's post-hoc tests, n=6-7 per group, all comparisons to mutant group. **p<0.001, *p<0.05). (B) The weight and femur length are reduced by 10-15% and 40%, respectively in *Rosa26*$^{mIfitm5/+}$;Prx1-Cre mutants (a summary of measurements in 2 months old females, one-way ANOVA with Tukey's post-hoc tests, n=6-7 per group, all comparisons to mutant group. *p< 0.05, **p<0.005, ***p<0.0005).

Figure S3

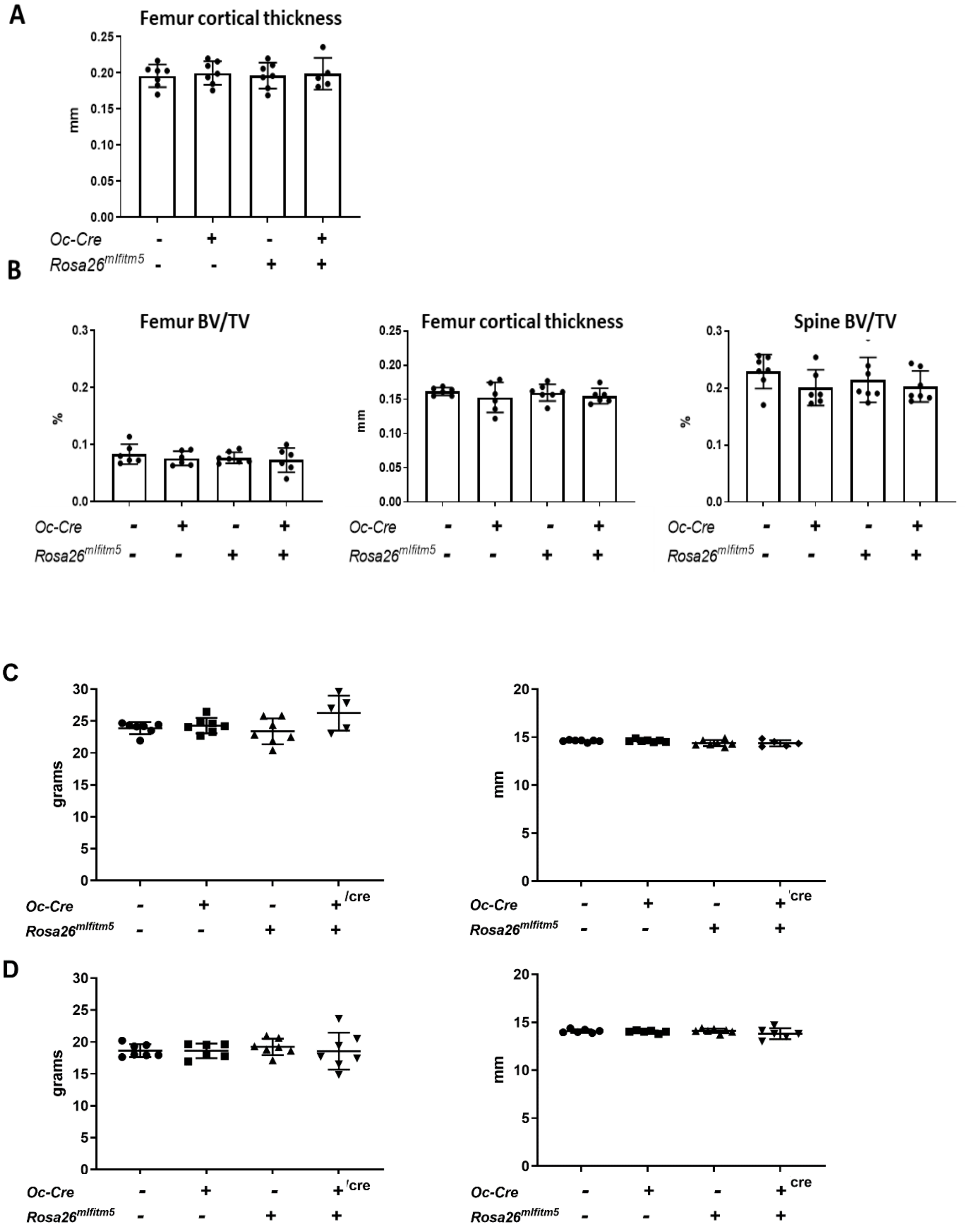
A
Femur cortical thickness
mm
0.25
0.20
0.15
0.10
0.05
0.00
Oc-Cre - + - +
Rosa26mIfitm5 - - + +
B
Femur BV/TV
%
0.3
0.2
0.1
0.0
Femur cortical thickness
mm
Spine BV/TV
Oc-Cre - + - +
Rosa26mIfitm5 - - + +
C
grams
30
25
20
15
10
5
0
mm
20
15
10
5
0
Oc-Cre - + - +
Rosa26mlfitm5 - - + +
D
grams
mm

Fig. S3: Growth and bone mass are not significantly altered in *Rosa26*$^{mIfitm5/+}$;OC-Cre mice. (A-B) Micro CT analysis showed no significant difference in bone architectural parameters in *Rosa26*$^{mIfitm5/+}$;OC-Cre mutants, including the femur cortical thickness, and bone volume/total volume (BV/TV) in femurs and spines. (C-D) The weight (left) and femur length (right) are not significantly different in *Rosa26*$^{mIfitm5/+}$*;*OC-Cre mutants. Analysis preformed in 2 months old males (A, C) and females (B, D) (One-way ANOVA with Tukey's post-hoc tests, n=5-7 per group, all comparisons to mutant group).

Figure S4

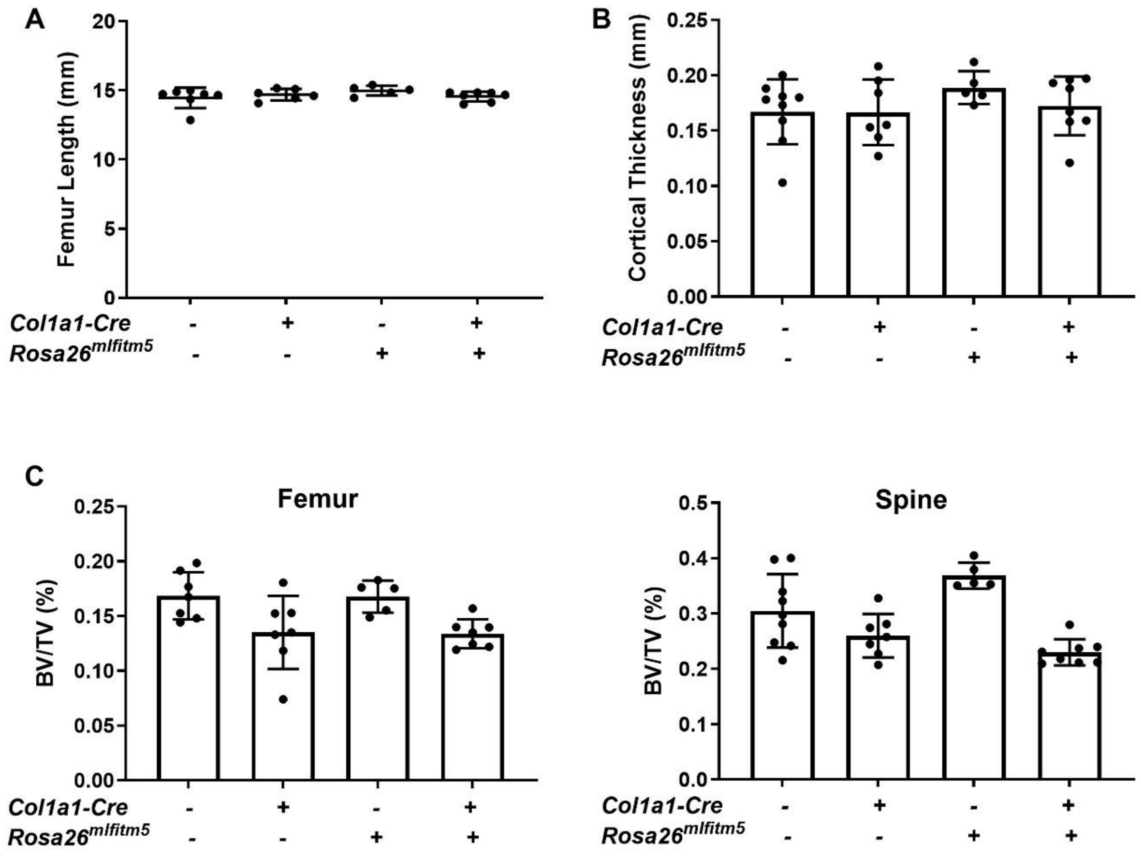

Fig. S4: Growth and bone mass are not significantly altered in *Rosa26*$^{mIfitm5/+}$;Col1a1-Cre mice. (A) Femur length is not significantly different in *Rosa26*$^{mIfitm5/+}$;Col1a1-Cre mutants. (B) Micro CT analysis showed no significant difference in the femur cortical thickness. (C) The bone volume/total volume (BV/TV) was mildly reduced in femurs and spines, but not significantly different than in Cre/- controls expressing only the Cre recombinase. Analysis preformed in 2 months old males (One-way ANOVA with Tukey's post-hoc tests, n=5-7 per group, all comparisons to mutant group).

Figure S5

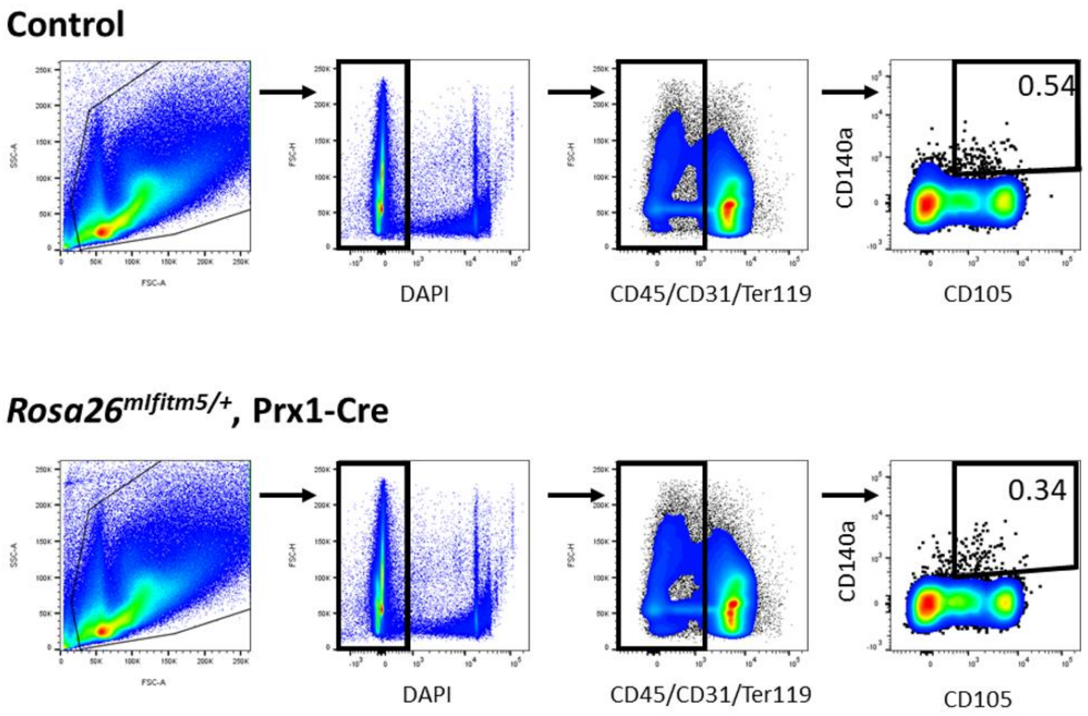

Fig. S5: Skeletal progenitor cell population is not significantly altered in the bone marrow in *Rosa26*$^{mIfitm5/+}$;Prx1-Cre mice. Flow cytometry analysis of skeletal progenitor markers in cells isolated from the bone marrow in control (top) compared to mutant (bottom).

Figure S6

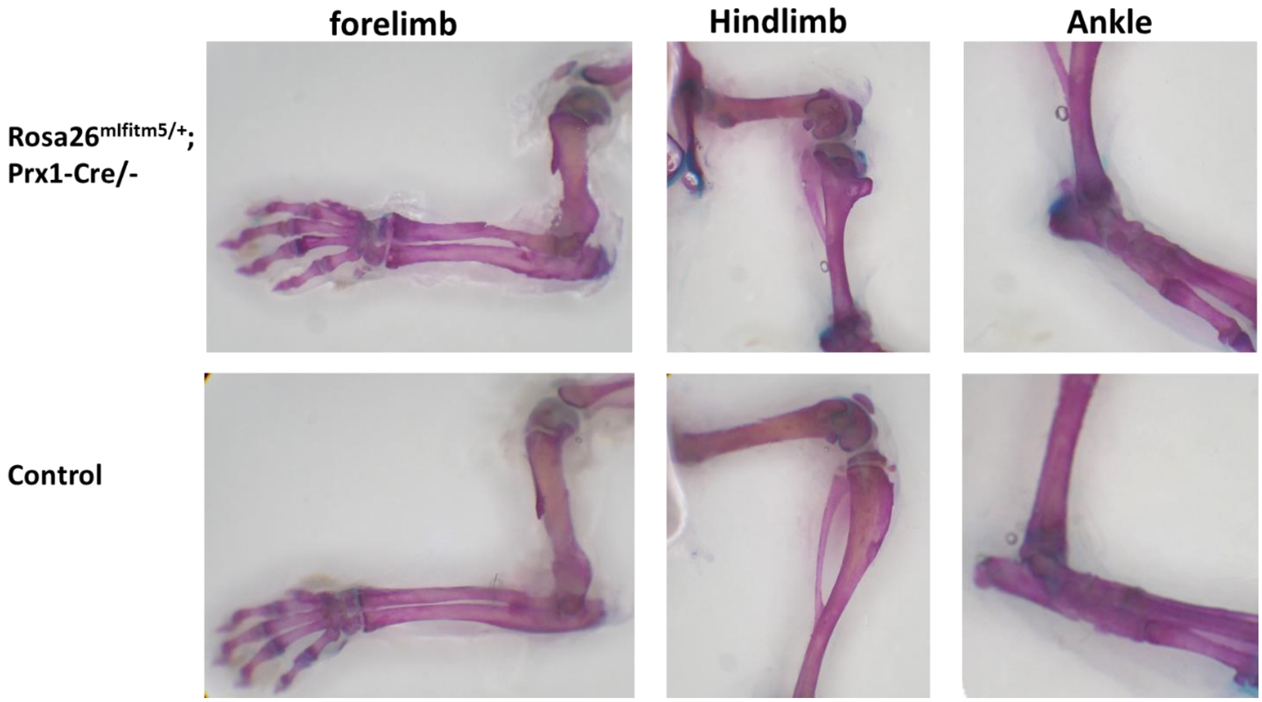

Fig. S6: Skeletal preparations demonstrate skeletal deformities in *Rosa26*$^{mIfitm5/+}$;Prx1-Cre mice. Skeletal preparations of 3 week old *Rosa26*$^{mIfitm5/+}$;Prx1-Cre mutant (top panel) and littermate control (bottom panel), showing shortening of long bones, joint deformities and partially mineralized cartilage overgrowth at the knees and ankles.

Figure S7

A

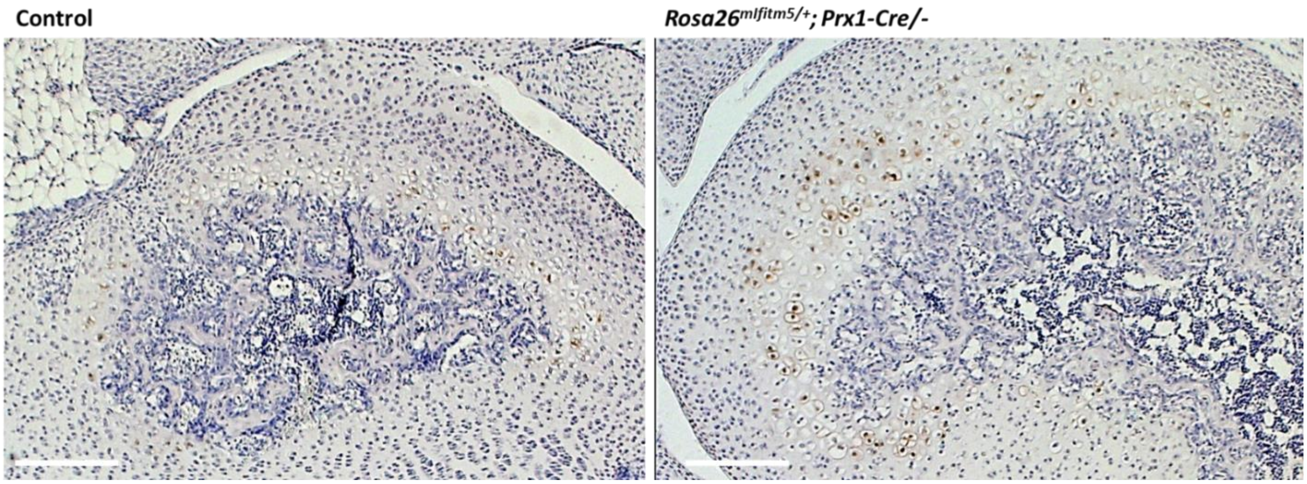
Control
Rosa26$^{mIfitm5/+}$; Prx1-Cre/-

B

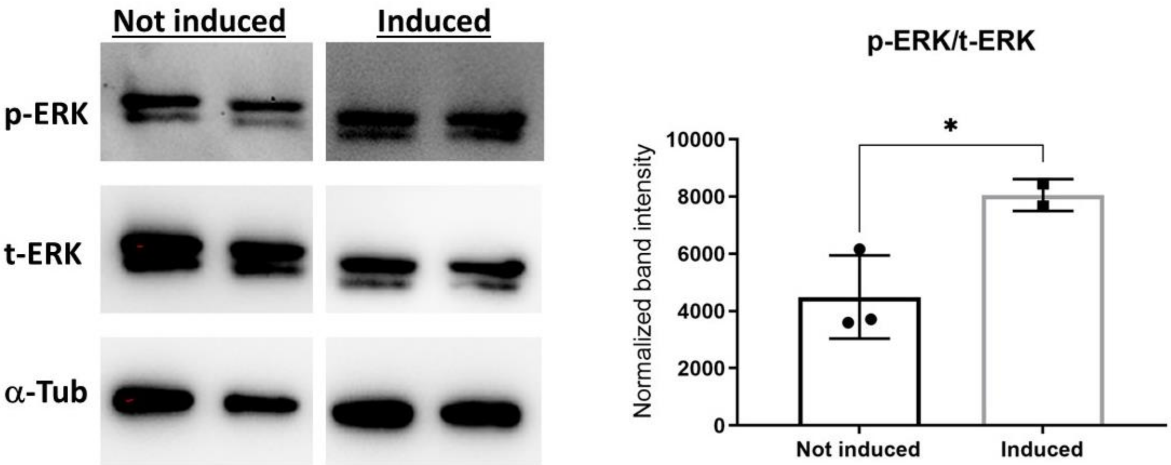
Not induced
Induced
p-ERK
t-ERK
α-Tub
p-ERK/t-ERK
*
Normalized band intensity
10000
8000
6000
4000
2000
0
Not induced
Induced

C

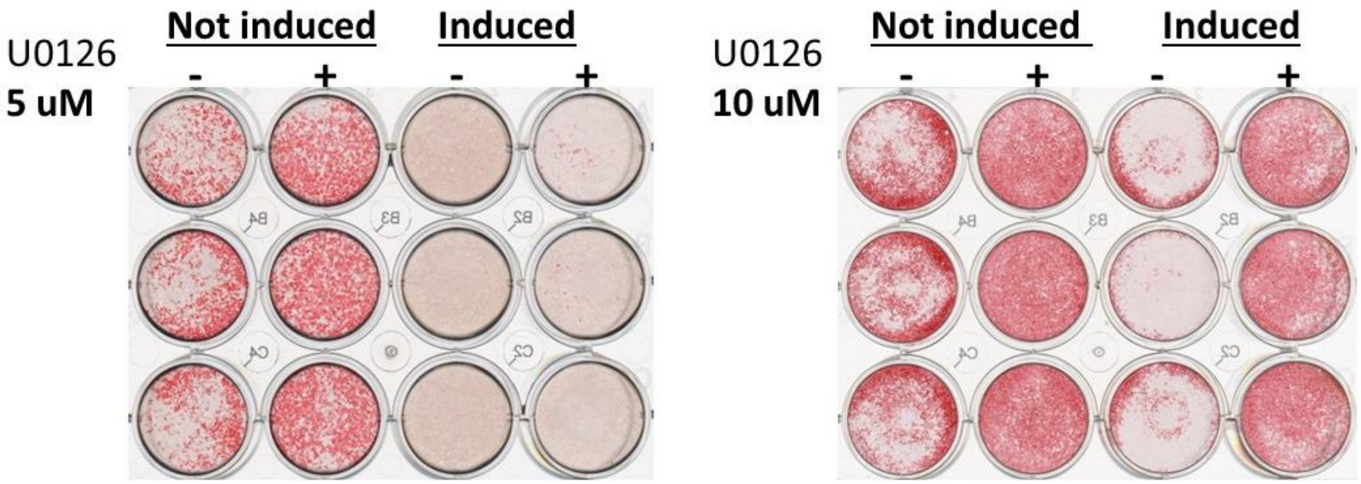

Fig. S7: ERK signaling is activated downstream of the IFITM5 mutation. (A) Immunohistochemistry staining for phosphorylated ERK in *Rosa26*$^{mIfitm5/+}$;Prx1-Cre mouse model. Representative image showing positive-stained chondrocytes (brown staining) adjacent to the secondary ossification site at the proximal tibia of mutant, but not in the wild type control (5X magnification, scale bar=200 um). (B) Increased ERK phosphorylation in MC3T3 cells expressing mutant IFITM5 under doxycycline induction (induced) compared to baseline (Not induced). Western blot (left panel) and quantification (right panel) of phospho-ERK/total ERK band intensity normalized to tubulin (t-test, *p=0.03). (C) Delayed mineralization in cells expressing mutant IFITM5 (right two columns-doxycycline induced mutant IFITM5 expression) was restored by treatment with the ERK inhibitor U0126 in a dose-dependent manner. Uninduced cells are in left two columns. Cells were incubated for 14 days in differentiation media containing ascorbic acid, beta glycerophosphate and BMP2, with either U0126 (5 uM or 10 uM) or vehicle (methanol).

Figure S8

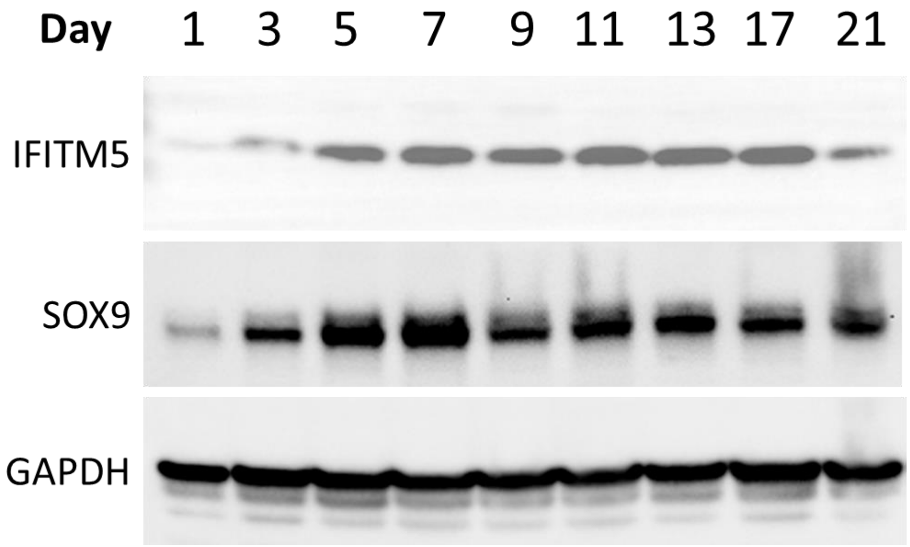

Fig. S8: IFITM5 expression in ATDC5 cells correlates with chondrogenic differentiation. IFITM5 and SOX9 protein expression are co-induced, as shown by western blot analysis and normalized to GAPDH. ATDC5 cells were incubated for 21 days in differentiation media containing insulin-transferrin-selenium (ITS) supplement.

Figure S9

A

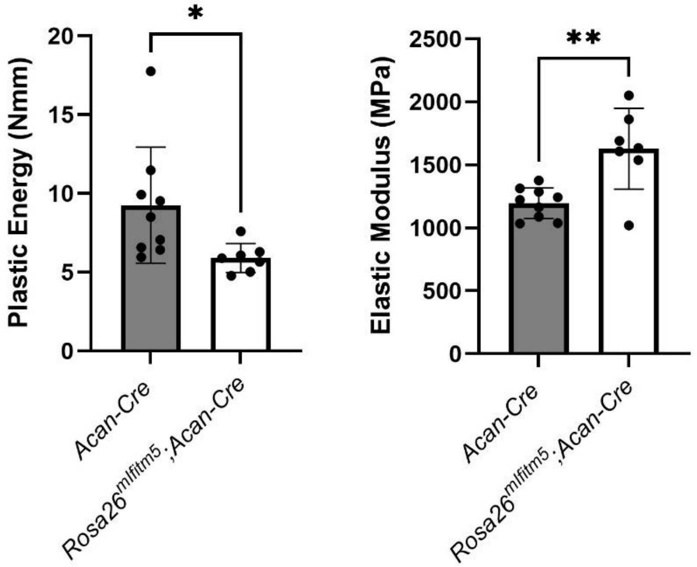

B

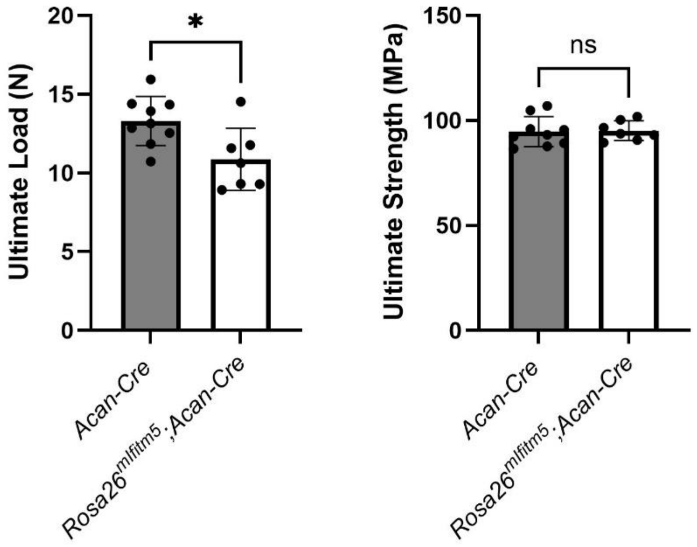

Fig. S9: Biomechanical testing of femurs in *Rosa*26^{mIfitm5};*Acan-Cre ERT2* mutant mice by 3-point bending. (A) Analysis demonstrated altered bone mechanical properties, including reduced ductility (plastic energy, left) and increased bone stiffness (elastic modulus, right) in the

mutant mice. (B) Bone strength (ultimate load, left) was reduced in the mutant mice, although when normalized to femur length (ultimate strength, right) there was no significant difference between mutant and control femurs. *Cre* recombinase was activated by intraperitoneal tamoxifen injections (10 mg/kg/dose) at P.10-15 and samples collected at age 5 weeks (t-test, n=7-9 per group, $^*p < 0.05$, $^{**}p < 0.005$, ns=not significant).

Figure S10

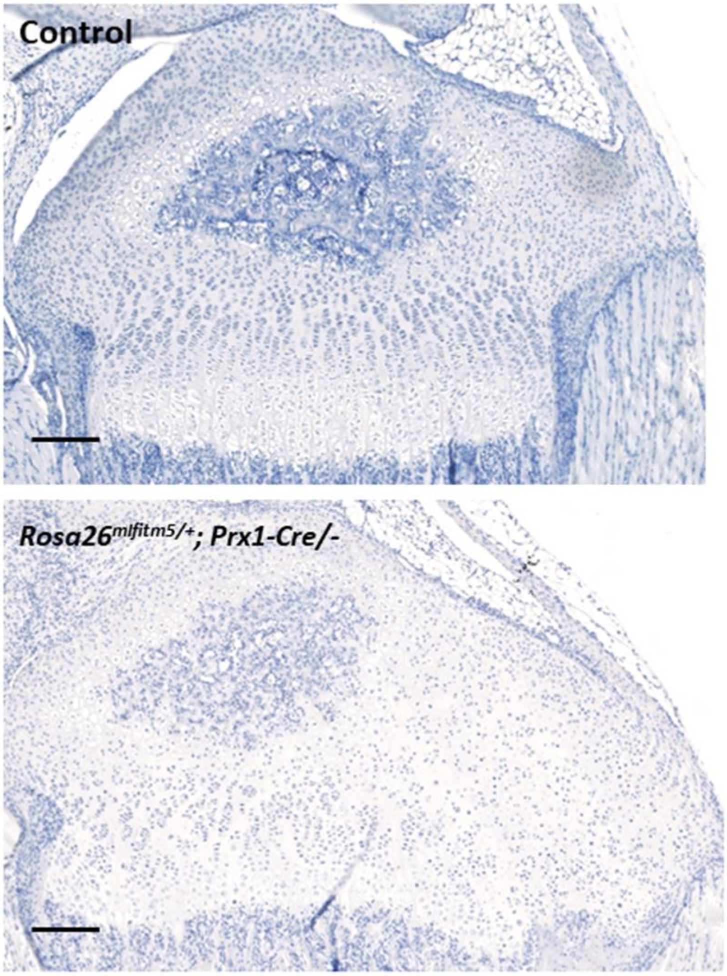

Fig. S10: Representative images of proximal tibia of control (top) and mutant (*Rosa26*$^{mIfitm5/+}$;Prx1-Cre, bottom) mice at age 2 weeks, immunohistochemistry without primary antibody (secondary only control, scale bar=200 um).

Figure S11

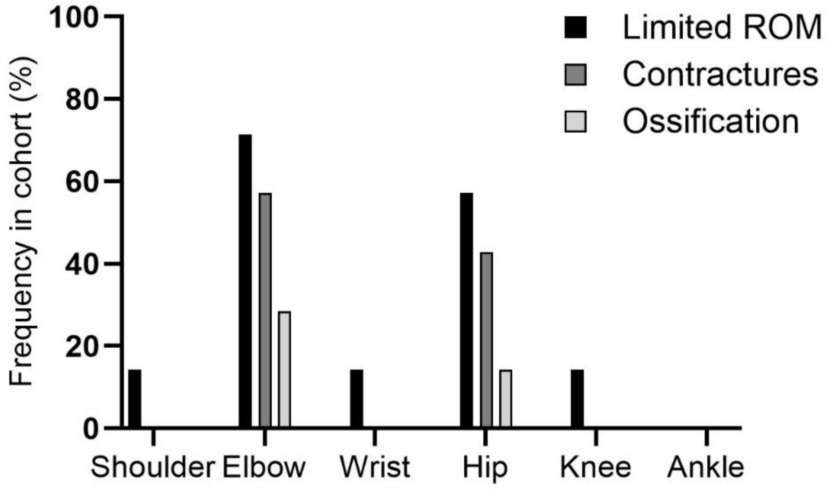

Fig. S11: Joint pathology in individuals with OI type V. We performed a retrospective chart review of seven individuals with OI type V from 3 skeletal centers in the United States (Table S1). All participants had a confirmed molecular diagnosis of OI type V. The frequency of joint phenotype in our study cohort (n=7) is presented here as a function of the joint involved and type of joint dysfunction. Variable degree of joint dysfunction was documented in six of the seven participants (85%). Most frequently, joint pathology manifested as limited range of motion by physical exam, which more commonly involved the elbows and hips. ROM = Range of Motion.

Figure S12

A

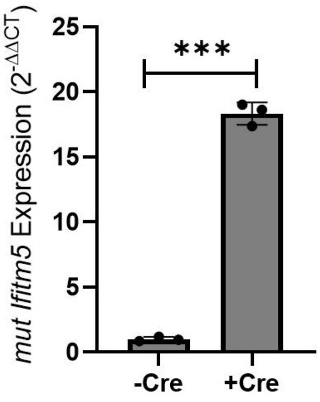

B

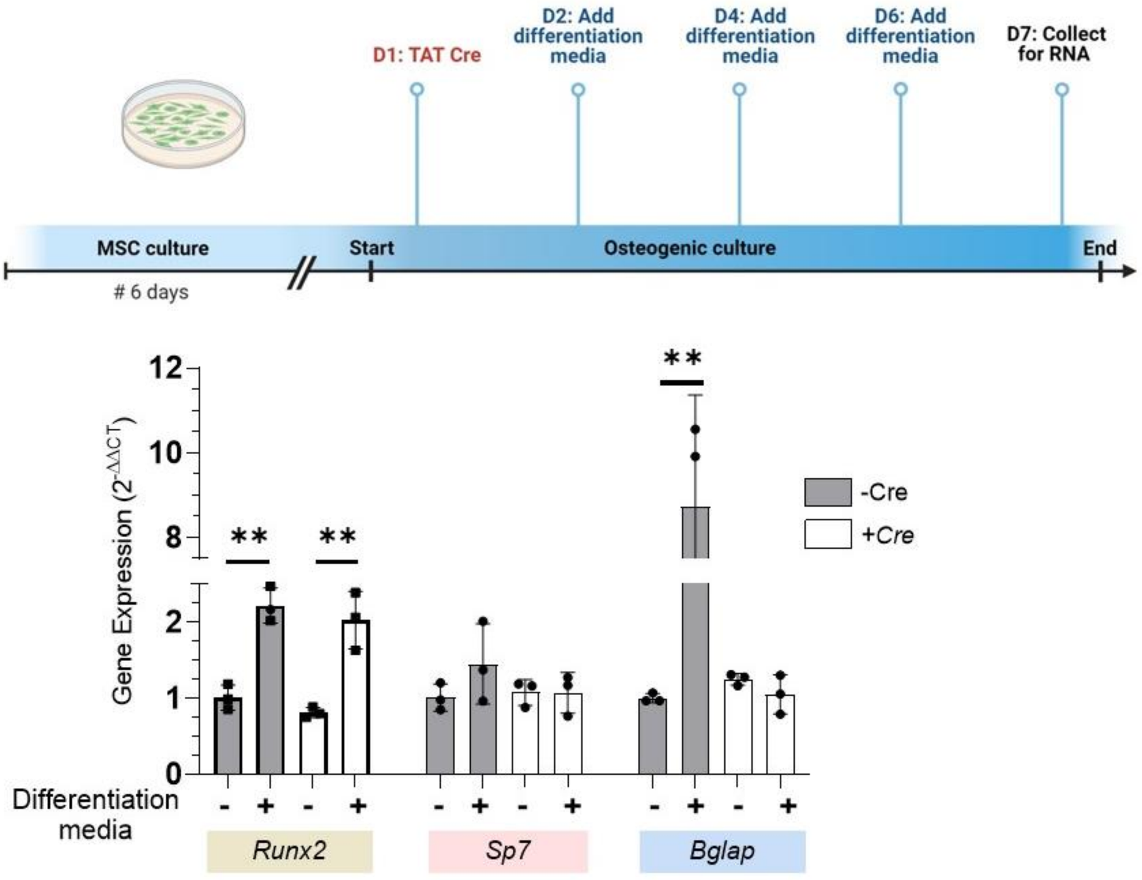

C

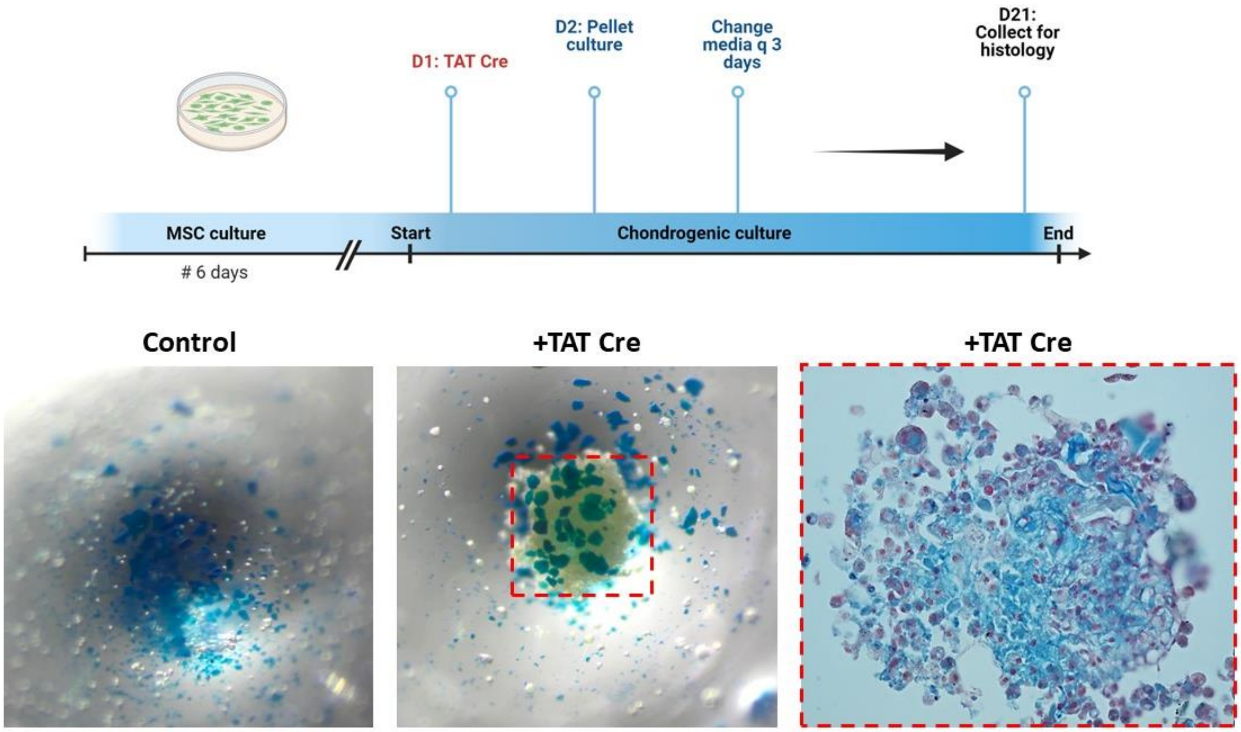

Fig. S12: In vitro differentiation of marrow stromal cells expressing the common OI type V mutation. (A) qPCR analysis showing the expression of mutant *Ifitm5* cDNA in primary marrow stromal cells (MSC) from *Rosa26*$^{mIfitm5/+}$ mice, with and without Cre recombinase treatment. (B) MSC differentiation to osteoblasts. Cells were incubated for 6 days in differentiation media containing ascorbic acid and beta glycerophosphate prior to RNA extraction. qPCR analysis shows up regulation of osteocalcin (*Bglap*) and a trend of increase in *Sp7* in control samples (-Cre), but not in cells expressing the mutant *Ifitm5* allele (+Cre). *Runx2* expression was up regulated in both groups. (C) MSC differentiation to chondrocytes. Cells were pellet-cultured and incubated for 21 days in chondrogenic media, then fixed and stained with Alcian blue. While single cells in both groups stained positive for Alcian blue, only cells expressing the mutant

Ifitm5 allele (+TAT Cre) condensed to form a cartilage-like structure (representative image from 2 independent experiments, n=3 technical repeats in each experiment). Bottom right panel shows a representative histology section of the condensation, stained with Alcian blue (counterstaining with Nuclear Fast Red). Real-time PCR analysis: n= 3 per group, ** $p<0.01$, *** $p<0.001$.

Table S1: Joint phenotype in subjects with OI type V

Subject ID	Age (years)	Sex	Calcification of IM	Hyperplastic Callus	Radial Head Dislocation	Decrease range of motion	Joint Contracture or Ankylosis	Ossification at the joint
**1**	14	F	No	Yes	No	Yes (Wrist, hip, knees)	No	No
**2**	20	F	Yes	Yes	Yes	Yes (Elbows, hips)	Yes (Elbows, hips)	Yes (Elbows)
**3**	15	F	Yes	No	Yes	Yes (Elbows)	Yes (Elbows)	No
**4**	5	M	Yes	No	N/A	No	No	No
**5**	9	F	Yes	Yes	No	Yes (Elbows, hips)	Yes (Elbows, hips)	Yes (Elbows)
**6**	53	M	Yes	Yes	Yes	Yes (Shoulders, elbows, hips)	Yes (Elbows, hips)	Yes (Hips)
**7**	37	F	Yes	No	N/A	Yes	No	No

						(Elbows)		

IM=interosseous membrane

Table S2: Primer sequences

ES cell screening primers	
5’ Rosa26 for	5’-GGCGGACTGGCGGGACTA-3’
5’ Rosa26 rev	5’-GGGACAGGATAAGTATGACATCATCAAGG-3’
Targeted insert for I	5’- GGAGCGGGAGAAATGGATATG-3’
Targeted insert for II	5’-GCGAAGAGTTTGTCCTCAACC-3’
Targeted insert rev	5’-AAAGTCGCTCTGAGTTGTTAT-3’
3’ Rosa26 for	5’- ATTCGGCTATGACTGGGCACAACA -3’
3’ Rosa26 rev	5’- AGATTCTCAGTGGCTCAACAAC -3’
Mouse genotyping primers	
Rosa26^{mIfitm5} for	5’-GGGGACAAGTTTGTACAAAAAAGCAGGCTTAATGGCGCTGGAACCCA -3’
Rosa26^{mIfitm5} rev	5’-GGGGACCACTTTGTACAAGAAAGCTGGGTTATCTGATCAGATCTTATCGTCGTCATCCT -3’
∆LoxP for	5’- GTCCAGGGTTTCCTTGATGA-3’
∆LoxP rev	5’- TTGCAGCCAGGATGTTGTAG-3’
Universal Cre for	5’-TCCAATTTACTGACCGTACACCAA-3’
Universal Cre rev	5’-CCTGATCCTGGCAATTTCGGCTA-3’
Real time PCR primers	
Rosa26^{mIfitm5} KI for	5’-CTTGCACCTGTCCAAGTTAG-3’
Rosa26^{mIfitm5} KI rev	5’- CGTCGTCATCCTTGTAATCC-3’
mGapdh for	5’ -GCAAGAGAGGCCCTATCCCAA -3’
mGapdh rev	5’-CTCCCTAGGCCCCTCCTGTTATT-3’
mB2M for	5’-GGTCTTTCTGGTGCTTGTC-3’
mB2M rev	5’-CGTATGTATCAGTCTCAGT-3’

h*IFITM5* cloning primers (for zebrafish)	
h*IFITM5* wild type cDNA forward	5'-TGGAATTCTGCAGATATGGACACGGCGTATCC-3'
h*IFITM5* mutant cDNA forward	5'- TGGAATTCTGCAGATATGGCGCTGGAACCCAT-3'
h*IFITM5* cDNA reverse	5'- GCCACTGTGCTGGATTCAGTCATAGTCCGCGTCATCAAACTTGGT-3'

B2M=beta 2 microglobulin

1
