# Supplementary material for: The IFITM5 mutation in osteogenesis imperfecta type V is associated with an ERK/SOX9-dependent osteoprogenitor differentiation defect
Source: J Clin Invest. 2024 Jun 17;134(15):e170369. doi: 10.1172/JCI170369 (PMC11290974; doi:10.1172/JCI170369)

Full unedited blot for Figure 6B

Rabbit phospho-ERK

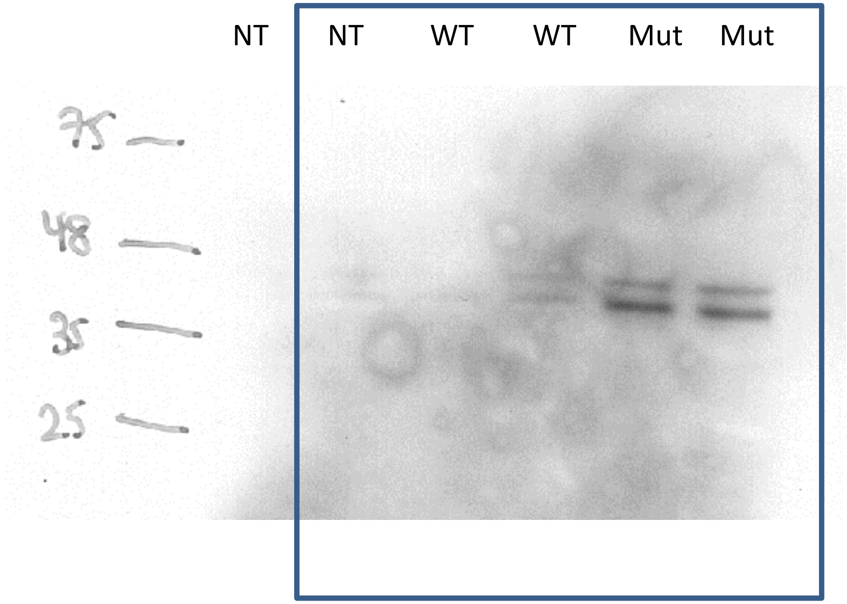

Rabbit total-ERK

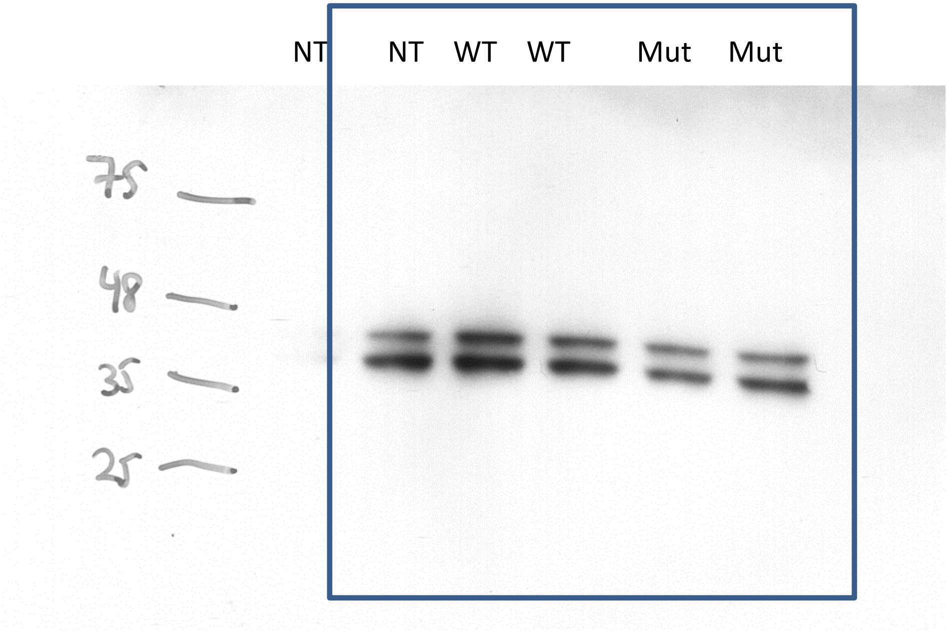

Full unedited blot for Figure S7 B

Rabbit phospho-ERK

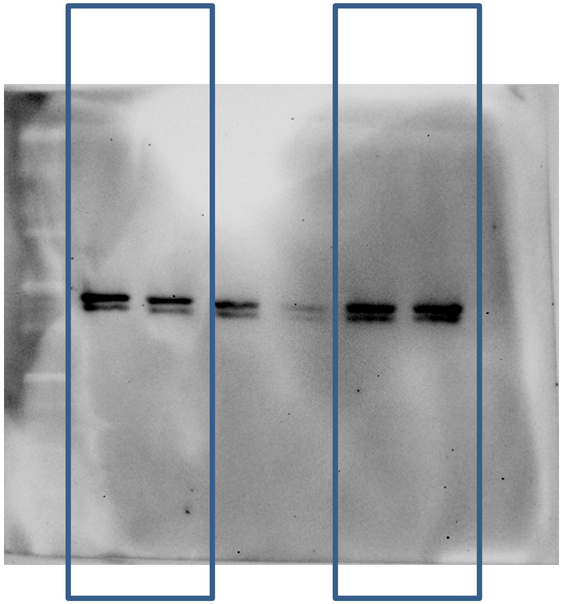

Rabbit total-ERK

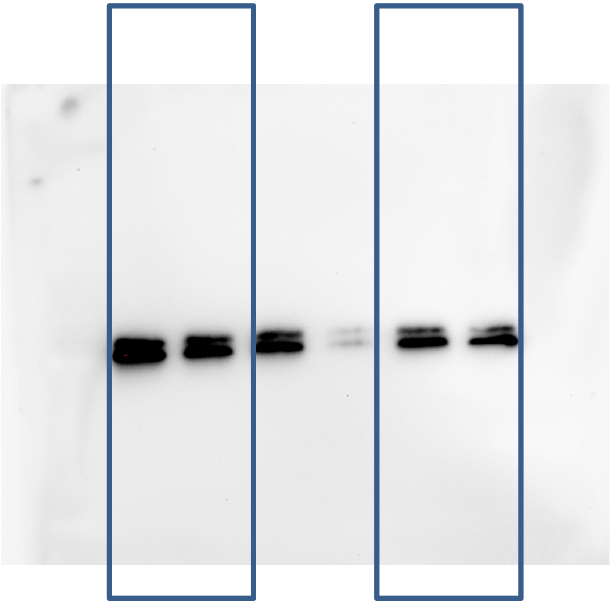

Tubulin

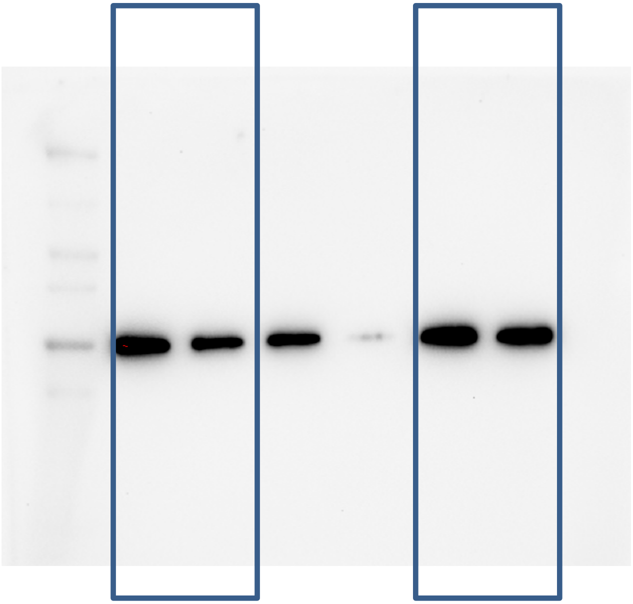

Supplement: Unedited blot and gel images [file jci-134-170369-s260.pdf]
